# Impact of the Tambora volcanic eruption of 1815 on islands and relevance to future sunlight-blocking catastrophes

**DOI:** 10.1038/s41598-023-30729-2

**Published:** 2023-03-04

**Authors:** Nick Wilson, Veronika Valler, Michael Cassidy, Matt Boyd, Lara Mani, Stefan Brönnimann

**Affiliations:** 1 Department of Public Health, University of Otago, Wellington, New Zealand; 2 Oeschger Centre for Climate Change Research, University of Bern, Bern, Switzerland; 3 School of Geography, Earth and Environmental Science, University of Birmingham, Birmingham, UK; 4 Department of Earth Sciences, University of Oxford, Oxford, UK; 5 Adapt Research Ltd, Reefton, New Zealand; 6 Centre for the Study of Existential Risk, University of Cambridge, Cambridge, UK; 7 Institute of Geography, University of Bern, Bern, Switzerland

**Keywords:** Climate sciences, Environmental social sciences, Natural hazards

## Abstract

Island nations may have potential long-term survival value for humanity in global catastrophes such as sun-blocking catastrophes from nuclear winter and large magnitude volcanic eruptions. One way to explore this issue further is to understand the impact on islands after the largest historically observed volcanic eruption: that of Mt Tambora in 1815. For each of the 31 large, populated islands selected, we conducted literature searches for relevant historical and palaeoclimate studies. We also analysed results from a reconstruction (EKF400v2), which uses atmospheric-only general circulation model simulations with assimilated observational and proxy data. From the literature review, there was widespread evidence for weather/climate anomalies in 1815–1817 for these islands (29/29 for those with data). But missing data was an issue for other dimensions such as impaired food production (seen in 8 islands out of only 12 with data). Based on the EKF400v2 reconstruction for temperature anomalies (compared to the relatively “non-volcanic” reference period of 1779 to 1808), the islands had lower temperature anomalies in the 1815–1818 period than latitudinally equivalent continental sites (at 100 km and 1000 km inland). This was statistically significant for the great majority of the comparisons for group analyses by hemisphere, oceans, and temperate/tropical zone. When considering just the islands, all but four showed statistically anomalous temperature reductions in the 1816–1817 period (for most p < 0.00001). In the peak impact year of 1816, the lowest anomalies were seen for islands in the Southern Hemisphere (p < 0.0001), the Indian Ocean (p < 0.0001), and in the tropics and subtropics of the Southern Hemisphere (p = 0.0057). In conclusion, the findings of both the literature review and reconstruction simulations suggest climatic impacts of the Tambora eruption for nearly all these 31 large islands, albeit less than for continental sites. Islands with the smallest temperature anomalies were in the Southern Hemisphere, in particular the Indian Ocean and the tropics and subtropics of the Southern Hemisphere.

## Introduction

The survival and flourishing of human civilisation could be threatened by an abrupt global catastrophe that reduced sunlight reaching the earth^1,2^. Such catastrophes include nuclear winter from a nuclear exchange^3^, a large magnitude volcanic eruption (magnitudes 7+ on the volcanic explosivity index) with stratospheric ejection^4^, and a large asteroid/comet impact^5^. The associated global climate impacts could include a drop in mean temperature, and a reduction in precipitation, that would limit food production, possibly causing a catastrophic global food shock^1^. Studies using climate models indicate that the impacts of catastrophes such as nuclear winter could be highly heterogeneous around the world^3,6–9^. For example, some of this work suggests that island nations in the Southern Hemisphere might be less affected than nations in Northern Hemisphere landmasses (e.g., Australia and New Zealand^10^). Similarly, large volcanic eruptions appear to be more common in the Northern Hemisphere, with one study of ice cores in Greenland and Antarctica indicating 60.2% of eruptions were in this hemisphere (n = 1113/1850) vs 39.8% in the Southern Hemisphere^11^.

Collectively the threat of these catastrophes are non-trivial, with estimates for the annual probability of inadvertent nuclear war being 1%^12^, or in the 0.3% to 3% range^13^. However, these could now be underestimates given the ongoing modernisation of some nuclear arsenals and with the Russian invasion of Ukraine in 2022. Eruptions of the Tambora scale in 1815 and larger (magnitudes 7 and 8+ on the volcanic explosivity index), occur around 1.6 times per 1000 years^11^, equivalent to around a one in six chance per century^14^. More probable are lower-magnitude (3–6) eruptions which might have “cascading, catastrophic effects” if they occur at critical pinch points where global critical systems converge e.g., marine shipping routes, submarine communication cables, and transportation networks^15^. An example was the Icelandic volcano (Eyjafjallajökull) that disrupted air transport in Europe in 2010^16^.

Some of these risks could be partially mitigated by reducing exposure and vulnerability to them globally e.g., de-alerting nuclear weapons and nuclear disarmament. For natural extreme risks, such as large magnitude volcanic eruptions and near-earth object impacts, prevention of the hazard currently remains improbable, with early warning and preparedness for civil protection remaining a last defence. In the worst-case scenario, where preparedness fails, humanity could benefit from having safe refuges to ensure continued human survival and to reboot technological civilisation^17,18^.

To this end, we therefore aimed to further explore the issue of sun-blocking catastrophes on potential island refuges by examining the impacts of the volcanic eruption of Mt Tambora in Indonesia in April 1815, the largest historically observed eruption^19^. This eruption cooled global land temperatures in 1816 by an estimated − 1.9 °C (± 0.2 °C)^20^, and contributed to famines in parts of Europe, India and China^21^. Indeed, the European summer of 1816 has been described as the “year without a summer”^22^, due to the extreme cold and wet conditions. Following this in 1817, some countries experienced the “year of famine”^23^.

The impact of this eruption has been given stronger support from a recent climate modelling study^24^. This work reported that “in climate models, including the forcing by the Tambora eruption makes the European cold anomaly up to 100 times more likely, while the precipitation anomaly became 1.5 and 3 times as likely, attributing a large fraction of the observed anomalies to the volcanic forcing”^24^. The impact of this eruption and 1816’s “year without a summer”, have also previously been used as a scenario to assess fragility of the global food trade system for wheat and rice^25^.

## Methods

### Island selection

We included the largest inhabited islands, using the minimum size criteria of at least 25,000 km^2^ in area^26^ and a minimum population size criteria of at least 100,000 people (in 2022). Both were arbitrary thresholds but were designed to make this study more relevant to considering islands with some potential capacity in terms of size and population to allow for being surviving “nodes of persisting complexity”^17^. We included Australia in our list of islands even though it is a “continental” island. Also included were islands that are jurisdictionally complex in the modern era, e.g., they have parts governed by separate nation states (e.g., the islands of: Borneo, Hispaniola, Ireland, Isla Grande de Tierra del Fuego, New Guinea, and Timor).

### Literature search

Literature searches were conducted during April to August 2022 using Google Scholar and the search term of “Tambora and 1815” and specific searches for each island using the search terms “Tambora and [island name/s]”. Historical studies of food prices and famines in each island were also searched for. Such searches were also conducted using Scopus and using “Elicit.org” (a digital research assistant for literature searches using an artificial intelligence system [GPT-3] and access to 175 million articles: https://elicit.org/faq#what-is-elicit). Specific island name searches were also conducted in the digital versions of five key texts, i.e., those by Harington^27^, Wood^28^, Brönnimann and Krämer^21^, Klingaman and Klingaman^29^, and Behringer^23^.

### Impact definitions

In terms of likely impacts of the Tambora eruption on islands in the 1815 to 1817 period, we considered weather/climate impacts to be those involving anomalous temperature and/or precipitation changes (as measured with instrumentation, documented by observers at the time, or from palaeoclimate studies e.g., of tree-rings or coral). For adverse food production impacts we defined these as where crop failures or reduced harvests were reported or where food prices rose. For adverse food insecurity impacts we defined these as reports of increased hunger, increased begging, and reported famines. We did not automatically assume that new epidemics (e.g., of typhus) reflected increased malnutrition, but documented the occurrence of these, given that they might reflect underlying malnutrition.

### Reconstructed climate

We included results from a climate reconstruction, EKF400v2^30^, which uses atmospheric-only general circulation model simulations (with sea-surface temperatures, land cover, and external forcings prescribed from reconstructions)^31^. The reconstruction estimates monthly climatological data for the 1600 and 2005 time period and builds on an earlier version (EKF400) published in 2017^32^. The EKF400v2 reconstruction has performed well in describing the Central European drought of 1726–1728 and provides insights into the climate dynamics leading up to this extreme dry period^30^. In another case study, it also performed well in reconstructing El Niño Southern Oscillation (ENSO) effects in the nineteenth century^30^. The earlier version of this reconstruction (EKF400), has also informed the impact of volcanic eruptions on the late phase of the Little Ice Age^33^.

Island-specific data inputs into the EKF400v2 reconstruction covering the time of the Tambora eruption were available for just over half of the islands (51.6%, 16/31). These were mainly from tree-ring studies (51.6%, 16/31), but also from instrumental records (9.7%, 3/31), and other sources (16.1%, 5/31; e.g., documentary sources and coral data). The mean number of data inputs per island was 1.1, range: 0 to 6 (see Table S1 in the Supplementary Information File 1 for details). But where such island-specific observational evidence was lacking, the reconstruction output was driven by the underlying model simulations.

In this analysis we focused on just the temperature anomalies given that these were statistically far more likely to be related to the volcanic forcing from Tambora, than precipitation anomalies (see “Introduction” section)^24^. For each island we used the reconstructed temperature data for a single geographic coordinate, the latitude and longitude for the most populous city on the island (listed in Table 4). In the reconstruction this single point reflects the results for a grid cell with dimensions of two degrees latitude and longitude square (approximately 222 km^2^ at the equator). The estimates presented in the results were for the temperature anomalies relative to the 1779 to 1808 period (as used in previously published work^33^). The latter was selected as the closest 30-year period which had no major known global volcanic forcing (i.e., there was the 1783 Laki eruption in Iceland, but this was largely tropospheric in its ejection pattern; and this period ended with a likely circa 1809 eruption of unknown location—see the “Discussion” section). Each annual result was the mean of the monthly anomalies for that year, with each monthly result being the ensemble mean of 30 model realisations.

### Comparisons of the islands with latitudinally-equivalent continental sites

To first ascertain the impact of the Tambora eruption on islands compared to continents, we compared the temperature anomalies for the islands relative to the “non-volcanic” reference period (of 1779 to 1808) with locations at the same latitude on the nearest continent (at 100 km inland and 1000 km inland). Where the nearest continental land mass was part of a peninsula, we chose the next nearest continent (relevant in three cases). Also, where the continent width was too narrow, we chose 500 km inland instead of 1000 km inland (relevant in two cases and in one case to avoid a large inland lake). One island was entirely excluded from the analysis (Isla Grande de Tierra del Fuego), as there is no continent on its latitude. The selected continental regions were: Africa (n = 11), Central America (2), East Asia (3), Europe (3), North America (2), Northern Asia (2), South America (4), Southeast Asia (2), and Western Asia (1). Fig. S1 in the Supplementary Information File 1 shows the specific continental sites for each island.

### Statistical analysis

In addition to the continental comparisons and the impacts on the individual islands, grouped analyses were conducted by: hemisphere, ocean, tropics/temperate zones. In the statistical analyses we used ANOVA or the Kruskal–Wallis test if the data was not normally distributed (if p < 0.05 on Bartlett’s test for inequality of population variances). Excel files of the reconstruction data are available in the Supplementary Information File 2 and File 3.

## Results

### Literature review findings

**Figure 1:**
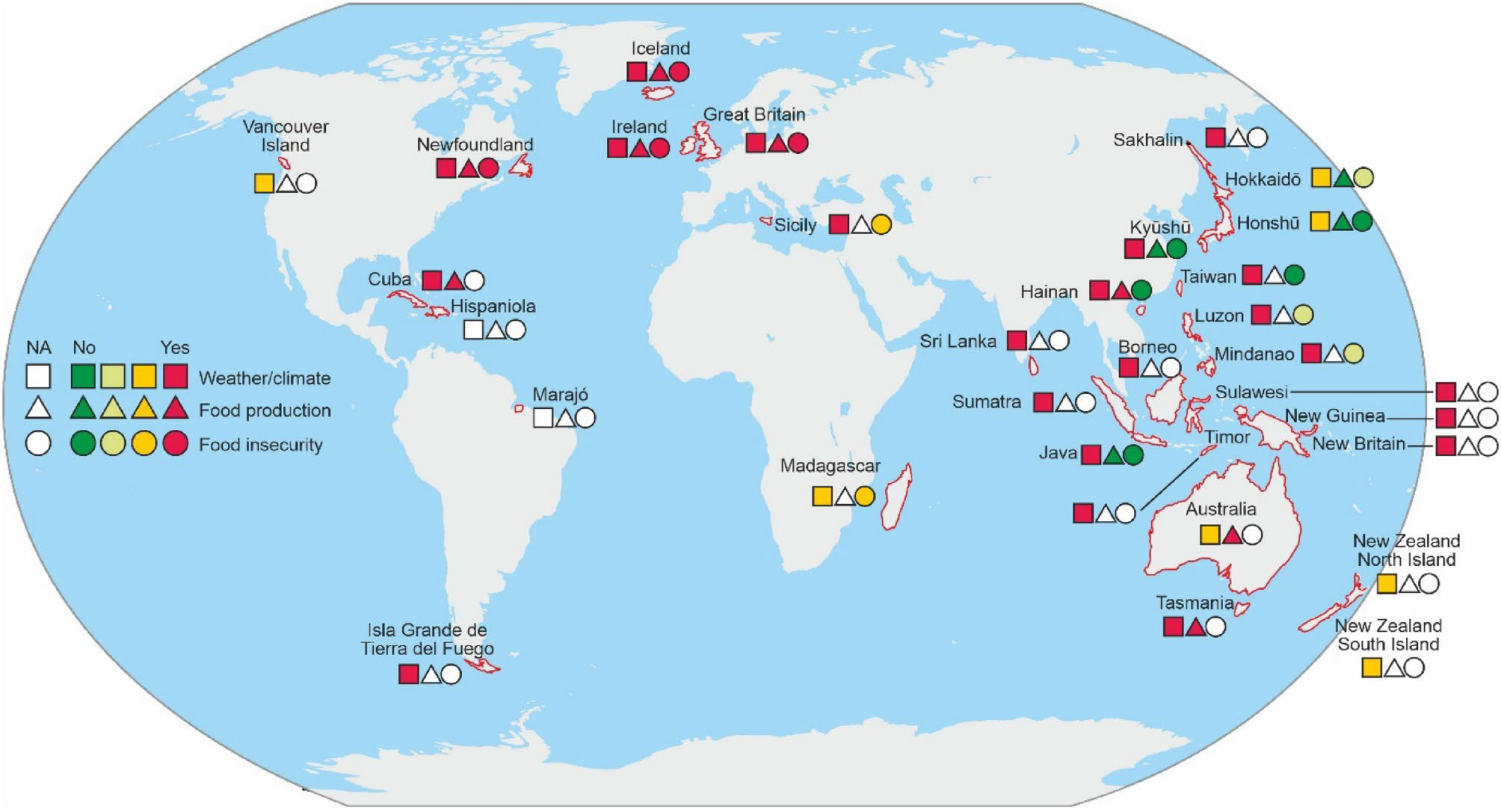
Summarised evidence for impacts from the Tambora eruption on 31 islands in the 1815–1817 period (see Table 1, Table S2 for additional details and assessments around data with some degree of uncertainty where intermediate levels of colouring are used (i.e., lighter green where the overall assessment was “probably no” impact, and orange where the overall assessment was “probably yes” impact); Image produced using Ferret v7.63).

Out of the 31 islands included in this study, island-specific impact data were identified for 94% (29/31) (Fig. 1, Table 1). The two islands lacking any such data were Hispaniola in the Caribbean (modern day Haiti and the Dominican Republic), and Marajó located in the mouth of the Amazon River in Brazil. Of those islands with impact data for the 1815–1817 period, all (100%; 29/29) had at least some evidence of anomalous weather/climate in terms of temperature or precipitation. However, for some islands this evidence was only rated as “probable impact” given some aspects of the mix of data being consistent with no weather/climate impact.

**Table 1:** Evidence for impacts on the weather/climate and food production/security (or not) from the Tambora eruption of 1815 on 31 islands for any of the years 1815–1817 (see Table S2 in the Supplementary Information File 1 for additional details and data sources for each island; cells with dashes indicate no relevant data identified).

Island (as in 1815–1817)	Anomalous weather/climate	Adverse impacts on food production	Food insecurity/famine
North Atlantic Ocean (includes Caribbean and Mediterranean)			
Cuba (then part of the Spanish Empire)	Yes	Yes	–
Great Britain (England, Scotland, Wales)	Yes	Yes	Yes
Hispaniola (now Haiti and Dominican Republic)	–	–	–
Iceland (then part of the Danish Empire)	Yes	Yes	Yes
Ireland (then in a union with Great Britain)	Yes	Yes	Yes
Newfoundland (then part of the British Empire, now part of Canada)	Yes	Yes	Yes
Sicily (then part of a kingdom of Southern Italy)	Yes	–	Probably yes (given an epidemic)
South Atlantic Ocean			
Isla Grande de Tierra del Fuego (now part of both modern-day Chile and Argentina)	Yes	–	–
Marajó (Brazil) (then part of the Portuguese Empire)	–	–	–
North Pacific Ocean			
Hainan (an island province of China)	Yes	Yes	No impact
Japan—Honshū	Probably yes*	No impact	No impact
Japan—Hokkaidō	Probably yes*	No impact	Probably no impact
Japan—Kyūshū	Yes	No impact	No impact
Philippines—Luzon (then part of the Spanish Empire)	Yes	–	Probably no impact
Philippines—Mindanao	Yes	–	Probably no impact
Sakhalin (part of modern-day Russia)	Yes	–	
Taiwan (a province of China in 1815–1817)	Yes	–	No impact
Vancouver Island (then part of the British Empire, now Canada)	Probably yes*	–	–
South Pacific Ocean			
Australia (the main island continent—data for New South Wales only)	Probably yes*	Yes	–
Australia—Tasmania (then named “van Diemen’s Land”)	Yes	Yes	–
Indonesia—Borneo (Kalimantan is the Indonesian portion of Borneo; other parts are East Malaysia and Brunei)**	Yes	–	–
Indonesia—Sulawesi (formerly “Celebes”)**	Yes	–	–
New Britain (part of modern-day Papua New Guinea [PNG])	Yes	–	–
New Guinea (now both part of modern-day PNG and part of Indonesia)	Yes	–	–
New Zealand—North Island (then part of the British Empire)	Probably yes*	–	–
New Zealand—South Island	Probably yes*	–	–
Indian Ocean			
Indonesia—Sumatra** (part of the British Empire until Dutch rule began again in 1816)	Yes	–	–
Indonesia—Java	Yes	No impact	No impact
Madagascar	Probably yes*	–	Probably yes (because of a reported famine, albeit detail lacking)
Sri Lanka (then part of the British Empire and called Ceylon)	Yes	–	–
Timor (part of modern East Timor and part of modern Indonesia)	Yes	–	–

*Mixed evidence but some impact probably dominated.

**These three islands actually straddle the equator so are in both the North and South Indian Ocean (Sumatra) and both the North and South Pacific (Borneo and Sulawesi).

Missing data was predominant for food production and insecurity impacts. Nevertheless, for islands with data, there was evidence of adverse impacts on food production (8 out of 12 islands with data). These islands were Cuba, Great Britain, Hainan (China), Ireland, Iceland, Newfoundland (Canada), part of the main continental island of Australia (New South Wales), and Tasmania (Australia). Islands with evidence for *no* impact on food production were only the Japanese islands of Hokkaidō, Honshū, and Kyūshū; and the Indonesian island of Java of Java (Table 1). The latter did experience some direct ash fall impacts from the eruption in 1815, but the share of the GDP for agricultural exports and for textile production was relatively stable for the 1815–1820 period (Fig. 10 in Ref.^34^). If there were substantial food shortages it would seem likely that exports would have declined. While rice consumption per capita and rice-growing area did decline (Fig. 2 in Ref.^34^)—this was part of a pattern for all of 1815 to 1830, and probably reflects other trends e.g., use of agriculture land for other food crops (e.g., maize, pulses and tubers) and for export crops (e.g., textiles, coffee, tea, tobacco, sugar and indigo).

Data on food insecurity or famines was also largely missing, but for the 12 islands with data, four definitely experienced such problems. These were all in the North Atlantic region i.e., Great Britain, Ireland, Iceland and Newfoundland. In three of these there was also evidence of food riots or demonstrations (i.e., all except Iceland). In Ireland there was also evidence of increased death rates from famine and/or famine-related disease. But there was less definitive evidence for Sicily where a typhus epidemic in 1817 could have reflected levels of malnutrition, and for Madagascar where the cause of famines in 1816 and onward could have been due to other causes (e.g., conflict).

### Comparisons of the islands with latitudinally-equivalent continental sites

The results (Table 2) indicate that larger continental temperature anomalies (larger temperature reductions relative to the reference period) occurred for all latitudinally-equivalent continental sites compared to the islands. While none of these differences were statistically significant for the 100 km inland continental sites, they were all highly statistically significant for the 1000 km inland continental sites.

**Table 2:** Mean temperature anomalies (°C) (minimum to maximum anomalies) for each year in the 1815 to 1818 period relative to the “non-volcanic” reference period (1779 to 1808) for the 30 islands with latitudinally-equivalent points on continents (at the 100 km and 1000 km points) and using monthly data from the reconstruction EKF400v2 (see “Methods” section for additional details).

Year	Islands temperature anomaly [A]	Continental temperature anomaly (100 km inland points) [B]	p-value (difference between columns [A] and [B])*	Continental temperature anomaly (1000 km inland points) [C]	p-value (difference between columns [A] and [C])*
1815	− 0.280 (− 3.1 to 2.4)	− 0.338 (− 3.0 to 2.8)	0.2373	− 0.503 (− 3.7 to 1.9)	< 0.0001
1816	− 0.391 (− 2.5 to 1.4)	− 0.517 (− 3.6 to 1.9)	0.2341	− 0.569 (− 5.4 to 3.9)	0.0002
1817	− 0.292 (− 2.6 to 3.1)	− 0.325 (− 5.8 to 3.7)	0.9628	− 0.467 (− 8.0 to 5.1)	0.0001
1818	− 0.208 (− 2.5 to 2.9)	− 0.262 (− 7.5 to 6.4)	0.0895	− 0.389 (− 9.1 to 6.6)	< 0.0001
All 4 years	− 0.293 (− 3.1 to 3.1)	− 0.360 (− 7.5 to 6.4)	0.0546	− 0.482 (− 9.1 to 6.6)	< 0.0001
1816–1817 (most severe 2 years)	− 0.341 (− 2.6 to 3.1)	− 0.421 (− 5.8 to 3.7)	0.3966	− 0.518 (− 8.0 to 5.1)	< 0.0001

*All using the Kruskal–Wallis test since the data were not normally distributed.

When considering the whole 1815 to 1818 period (Table 3), the same pattern of larger temperature anomalies for the continental sites than the islands was also present. This was statistically significant for all but one of the comparisons between islands and the 1000 km sites (for both hemispheres, for the three main oceans, for five of the six oceans by hemisphere, and for both the temperate region and the tropical and subtropical region). For the 100 km sites, all but one of the equivalent comparisons involved larger temperature anomalies than the islands, and of these six were statistically significant. Furthermore, all but one of the mean anomalies at the 100 km site were less than those at the 1000 km sites (when considering all the results in Tables 2, 3).

**Table 3:** Mean temperature anomalies (°C) (minimum to maximum anomalies) for the 1815 to 1818 period relative to the “non-volcanic” reference period (1779 to 1808) for islands by location and the latitudinally-equivalent points on continents (at the 100 km and 1000 km points) and using monthly data from the reconstruction EKF400v2 (see “Methods” section for additional details).

Characteristic	Number of islands	Total number of monthly observations (on islands or each continental sites)	Islands temperature anomaly [A]	Continental temperature anomaly (100 km inland points) [B]	p-value (difference between columns [A] and [B])*	Continental temperature anomaly (1000 km inland points) [C]	p-value (difference between columns [A] and [C])*
Hemispheres**							
Northern Hemisphere (NH)	17	816	− 0.335 (− 3.1 to 3.1)	− 0.473 (− 7.5 to 6.4)	< 0.0001	− 0.595 (− 9.1 to 6.6)	< 0.0001
Southern Hemisphere (SH)	10	480	− 0.224 (− 0.8 to 1.1)	− 0.203 (− 2.3 to 1.5)	0.0207	− 0.328 (− 1.7 to 1.0)	< 0.0001
Main oceans spanning both hemispheres							
Atlantic Ocean***	8	384	− 0.323 (− 3.1 to 3.1)	− 0.402 (− 7.5 to 6.4)	0.1217	− 0.502 (− 9.1 to 6.6)	0.0112
Pacific Ocean	17	816	− 0.312 (− 2.3 to 1.8)	− 0.380 (− 3.1 to 2.3)	0.3275	− 0.504 (− 2.8 to 2.6)	< 0.0001
Indian Ocean^#^	5	240	− 0.180 (− 0.7 to 0.4)	− 0.226 (− 0.6 to 0.3)	0.0005	− 0.375 (− 1.0 to 0.4)	< 0.0001
Oceans by hemisphere**							
North Atlantic Ocean***	7	336	− 0.375 (− 3.1 to 3.1)	− 0.454 (− 7.5 to 6.4)	0.0782	− 0.563 (− 9.1 to 6.6)	0.0026
North Pacific Ocean	9	432	− 0.324 (− 2.3 to 1.8)	− 0.504 (− 3.1 to 2.3)	< 0.0001	− 0.654 (− 2.8 to 2.6)	< 0.0001
North Indian Ocean	1	48	− 0.149 (− 0.5 to 0.2)	− 0.326 (− 0.6 to − 0.1)	< 0.0001 (ANOVA)	− 0.288 (− 0.8 to 0.3)	0.0011 (ANOVA)
South Atlantic Ocean	1	48	+ 0.041 (− 0.7 to 1.1)	− 0.038 (− 0.8 to 1.0)	0.3689 (ANOVA)	− 0.075 (− 0.7 to 0.7)	0.1325
South Pacific Ocean	6	288	− 0.278 (− 0.8 to 0.3)	− 0.224 (− 2.3 to 1.5)	0.0003	− 0.337 (− 1.7 to 1.0)	0.0110
South Indian Ocean	3	144	− 0.206 (− 0.7 to 0.2)	− 0.217 (− 0.6 to 0.3)	0.5218 (ANOVA)	− 0.394 (− 1.0 to 0.2)	< 0.0001
Tropics and subtropics vs temperate zones^##^							
Tropics and subtropics	18	864	− 0.266 (− 1.8 to 1.1)	− 0.318 (− 2.5 to 1.0)	0.0032	− 0.438 (− 2.8 to 0.7)	< 0.0001
Temperate zone	12	576	− 0.333 (− 3.1 to 3.1)	− 0.425 (− 7.5 to 6.4)	0.3455	− 0.549 (− 9.1 to 6.6)	< 0.0001

*Using the Kruskal–Wallis test since the data were not typically normally distributed, unless indicated otherwise.

**Excluding the three islands that straddle the equator: Borneo, Sulawesi, and Sumatra.

***The Atlantic and North Atlantic groupings included islands in the Caribbean (Cuba and Hispaniola) and in the Mediterranean (Sicily).

^#^Including Java, Madagascar, Sri Lanka, Sumatra, and Timor. Although Australia borders both the Indian and Pacific Oceans, we classified it as in the South Pacific for this analysis.

^##^As per this map of the tropics, subtropics and temperate zones: https://commons.wikimedia.org/wiki/File:World_map_indicating_tropics_and_subtropics.png (that is, the following islands were included in the subtropical zone: Taiwan, the Japanese island of Kyūshū, and Australia (main continental island which is mainly in the tropics and subtropics, with a smaller southern part in the temperate zone).

In terms of maximal temperature anomalies, there was also greater cooling for the continental sites relative to the islands. This was the case for three of the four years for the comparison with the 100 km continental sites, and all four years for the 1000 km continental sites (Table 2). The same pattern of greater cooling in the continental sites was present for both hemispheres, for the three main oceans, for the six oceans by hemisphere, and for both the temperate region and the tropical and subtropical region (with minor exceptions being for the Indian Ocean and South Atlantic Ocean, Table 3). The largest such differences were for the North Atlantic Ocean (e.g., a maximal − 3.1 °C cooling for the islands vs − 7.5 °C at the 100 km sites and − 9.1 °C at the 1000 km sites).

### Reconstructed temperature anomalies for the islands and island groupings

**Figure 2:**
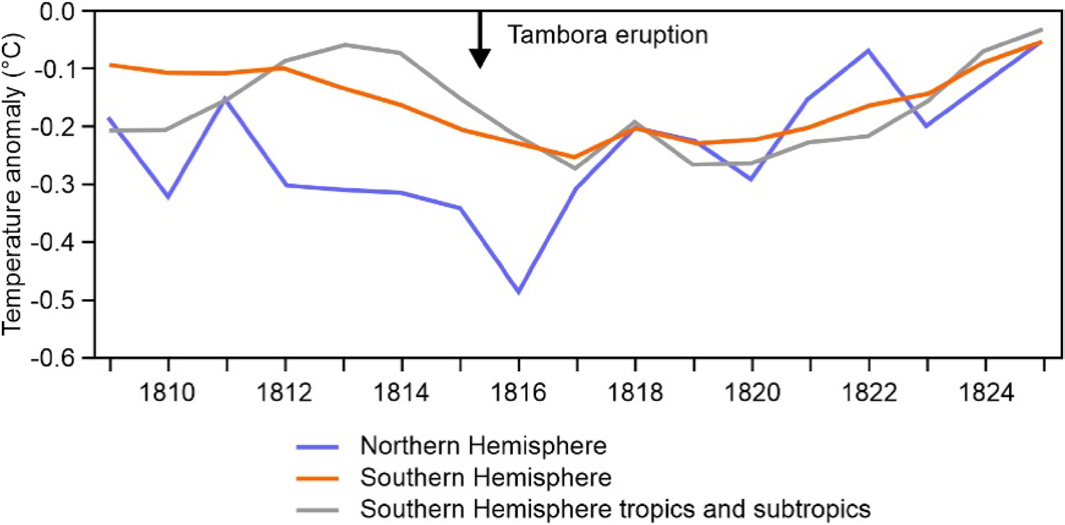
Reconstructed mean temperature anomalies relative to the “non-volcanic” reference period (1779 to 1808) using monthly data from the reconstruction EKF400v2 for the islands in this study by hemisphere/tropical zone (excluding the three islands that straddle the equator).

**Figure 3:**
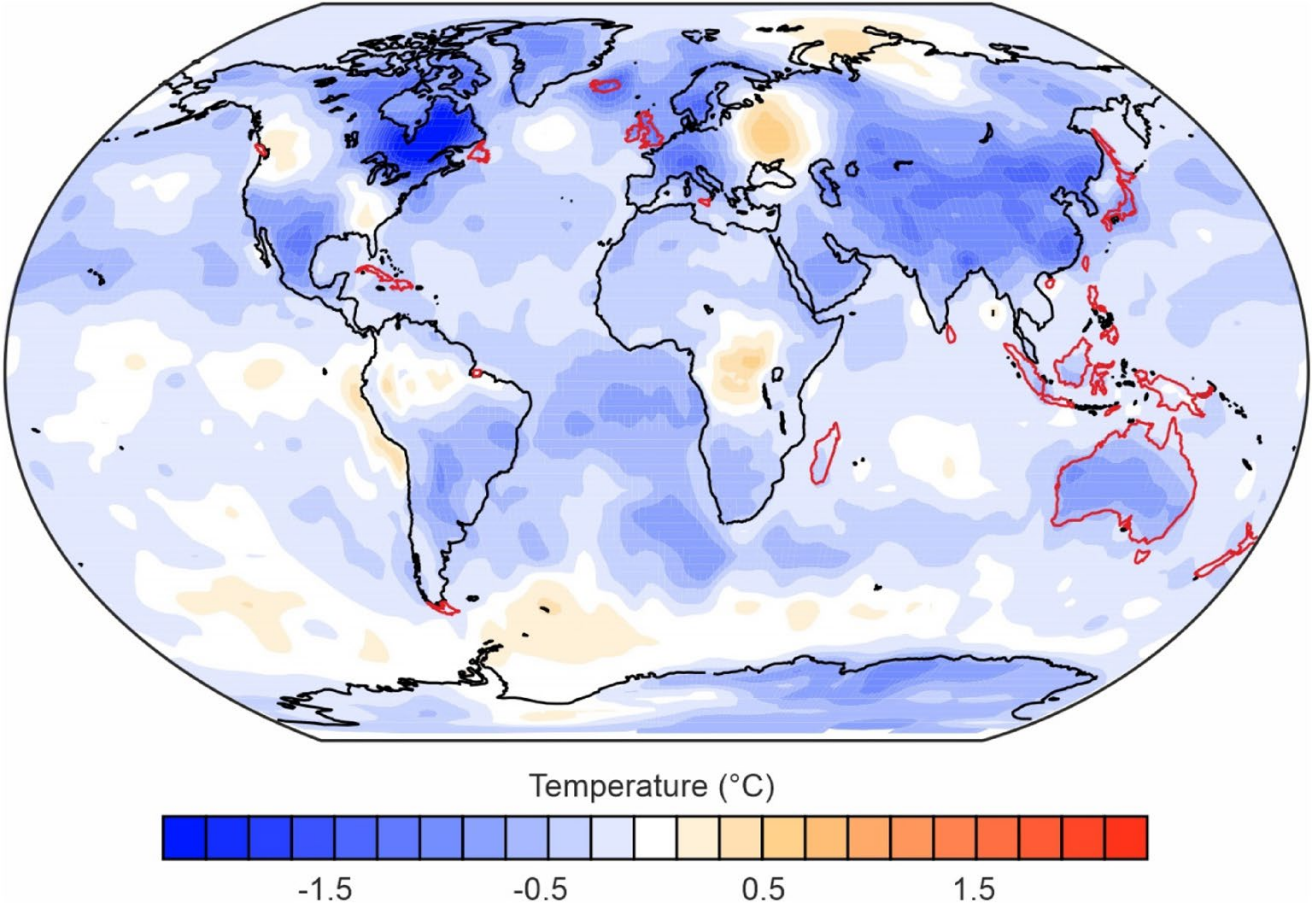
Reconstructed temperature anomalies in 1816 (“the year without a summer”) relative to the “non-volcanic” reference period (1779 to 1808) using monthly data from the reconstruction EKF400v2 (Image produced using Ferret v7.63).

Figure 2 gives the overall picture of the reconstructed temperature anomalies for islands in both hemispheres and for the tropics and subtropics in the Southern Hemispheres in the early 1800s. The mean temperatures were already lower than the reference period (1779 to 1808) in both hemispheres in 1809, but these declined further in 1815 and 1816. The decline and the overall anomaly was greatest in 1816 for the Northern Hemisphere. Figure 3 shows the temperature anomalies globally for this 1816 year.

As detailed in Table 4 below, all but one island had negative temperature anomalies for monthly means in 1815, compared to the reference period. The negative anomalies were largest for Honshū (− 0.80 °C) and were positive for Marajó (0.23 °C). The next largest negative anomalies were for Kyūshū (− 0.75 °C) and then Iceland (− 0.69 °C).

**Table 4:** Reconstructed anomalous temperatures (EKF400v2) with mean (minimum to maximum) monthly temperatures (°C) for each year for various time periods relative to the reference “non-volcanic period” of 1779 to 1808 (with each monthly temperature being the ensemble mean of 30 model realisations).

Island (with specific locality [city] used for the geographic coordinates)	1700 to 1778 (prior to reference period)	1779 to 1808 (non-volcanic reference period)	1815 (Tambora erupts)	1816	1817	1818	1816 to 1817 (peak Tambora period)	1819 to 1899 (rest of 1800s)	Peak vs reference period, p-value (ANOVA-unless stated)
North Atlantic Ocean (includes Caribbean and Mediterranean)									
Cuba [Havana]	− 0.068 (− 0.9 to 0.6)	0.0 (− 0.5 to 0.5)	− 0.269 (− 0.4 to − 0.1)	− 0.201 (− 0.4 to 0.1)	− 0.259 (− 0.5 to − 0.1)	− 0.266 (− 0.4 to − 0.1)	− 0.23 (− 0.5 to 0.1)	− 0.039 (− 1.4 to 1.3)	< 0.00001
Great Britain [London]	0.029 (− 5.3 to 3.9)	0.0 (− 5.8 to 4.6)	− 0.141 (− 3.1 to 2.4)	− 1.122 (− 2.5 to 0.3)	− 0.091 (− 2.6 to 3.1)	0.695 (− 0.9 to 2.9)	− 0.607 (− 2.6 to 3.1)	0.135 (− 6.2 to 4.6)	0.0552
Hispaniola [Port-au-Prince]	− 0.149 (− 0.6 to 0.2)	0.0 (− 0.4 to 0.4)	− 0.444 (− 0.5 to − 0.3)	− 0.503 (− 0.6 to − 0.4)	− 0.547 (− 0.6 to − 0.4)	− 0.524 (− 0.6 to − 0.4)	− 0.525 (− 0.6 to − 0.4)	− 0.165 (− 0.6 to 0.6)	< 0.0001 (KW*)
Iceland [Reykjavik]	− 0.166 (− 2.0 to 1.4)	0.0 (− 1.5 to 1.8)	− 0.694 (− 1.5 to − 0.2)	− 1.329 (− 2.0 to − 0.8)	− 0.942 (− 2.0 to 0.2)	− 0.557 (− 2.5 to 0.8)	− 1.135 (− 2.0 to 0.2)	− 0.301 (− 5.1 to 3.6)	< 0.00001
Ireland [Dublin]	− 0.01 (− 3.0 to 2.8)	0.0 (− 3.0 to 2.8)	− 0.155 (− 1.9 to 1.3)	− 0.717 (− 1.6 to 0.0)	− 0.154 (− 2.0 to 2.0)	0.314 (− 1.0 to 2.2)	− 0.436 (− 2.0 to 2.0)	0.052 (− 4.7 to 3.0)	0.0140
Newfoundland [St Johns]	− 0.087 (− 0.8 to 0.6)	0.0 (− 0.8 to 0.9)	− 0.246 (− 0.7 to 0.2)	− 0.173 (− 0.5 to 0.6)	− 0.424 (− 0.7 to − 0.1)	− 0.34 (− 1.2 to 0.2)	− 0.298 (− 0.7 to 0.6)	− 0.08 (− 4.0 to 2.1)	< 0.0001 (KW)
Sicily [Palermo]	− 0.095 (− 1.3 to 1.3)	0.0 (− 1.1 to 1.0)	− 0.336 (− 0.8 to 0.1)	− 0.536 (− 1.0 to − 0.3)	− 0.335 (− 0.8 to 0.1)	− 0.208 (− 0.4 to 0.3)	− 0.436 (− 1.0 to 0.1)	− 0.065 (− 1.5 to 1.3)	< 0.00001
South Atlantic Ocean									
Isla Grande de Tierra del Fuego [Ushuaia]	− 0.168 (− 2.4 to 0.9)	0.0 (− 1.6 to 1.0)	− 0.157 (− 0.7 to 0.5)	− 0.093 (− 0.6 to 0.8)	− 0.162 (− 0.9 to 1.0)	− 0.427 (− 1.8 to 0.2)	− 0.127 (− 0.9 to 1.0)	− 0.087 (− 2.2 to 1.7)	0.0717
Marajó [Breves]	− 0.064 (− 1.0 to 1.2)	0.0 (− 0.8 to 0.8)	0.233 (− 0.6 to 1.1)	− 0.014 (− 0.6 to 0.5)	− 0.176 (− 0.7 to 0.4)	0.122 (− 0.4 to 0.8)	− 0.095 (− 0.7 to 0.5)	0.274 (− 1.2 to 2.5)	0.1123
North Pacific Ocean									
Hainan [Haikou]	− 0.085 (− 0.6 to 0.4)	0.0 (− 0.3 to 0.3)	− 0.085 (− 0.3 to 0.2)	− 0.072 (− 0.3 to 0.1)	− 0.128 (− 0.4 to 0.2)	− 0.113 (− 0.3 to 0.1)	− 0.1 (− 0.4 to 0.2)	0.054 (− 0.6 to 1.0)	0.00001
Japan—Honshū [Tokyo]	− 0.151 (− 1.6 to 1.7)	0.0 (− 1.8 to 1.0)	− 0.804 (− 1.4 to 0.1)	− 0.909 (− 1.2 to − 0.3)	− 0.555 (− 1.3 to 1.1)	− 0.411 (− 1.3 to 0.7)	− 0.732 (− 1.3 to 1.1)	− 0.143 (− 2.3 to 2.2)	< 0.00001
Japan—Hokkaidō [Sapporo]	− 0.084 (− 0.8 to 0.8)	0.0 (− 0.8 to 0.6)	− 0.456 (− 0.7 to − 0.2)	− 0.454 (− 0.8 to − 0.2)	− 0.284 (− 0.7 to 0.1)	− 0.105 (− 0.5 to 0.1)	− 0.369 (− 0.8 to 0.1)	− 0.003 (− 2.2 to 1.8)	< 0.00001
Japan—Kyūshū [Fukuoka]	− 0.112 (− 1.2 to 1.5)	0.0 (− 1.1 to 1.3)	− 0.752 (− 1.8 to − 0.3)	− 0.713 (− 1.3 to − 0.3)	− 0.508 (− 1.1 to 0.0)	− 0.444 (− 1.0 to 0.0)	− 0.611 (− 1.3 to 0.0)	− 0.106 (− 2.4 to 2.5)	< 0.00001
Philippines—Luzon [Quezon City]	− 0.072 (− 0.3 to 0.2)	0.0 (− 0.2 to 0.3)	− 0.197 (− 0.3 to − 0.1)	− 0.214 (− 0.4 to − 0.1)	− 0.213 (− 0.3 to 0.0)	− 0.198 (− 0.3 to − 0.1)	− 0.213 (− 0.4 to 0.0)	− 0.038 (− 0.5 to 0.5)	< 0.00001
Philippines—Mindanao [Davao City]	− 0.115 (− 0.6 to 0.7)	0.0 (− 0.5 to 0.5)	− 0.102 (− 0.3 to 0.1)	− 0.191 (− 0.4 to 0.0)	− 0.21 (− 0.3 to 0.1)	− 0.195 (− 0.4 to 0.0)	− 0.2 (− 0.4 to 0.1)	0.027 (− 0.7 to 0.9)	< 0.00001
Sakhalin [Yuzhno-Sakhalinsk]	− 0.06 (− 0.9 to 1.0)	0.0 (− 1.1 to 0.8)	− 0.286 (− 0.8 to 0.2)	− 0.413 (− 1.0 to 0.0)	− 0.208 (− 0.7 to 0.3)	− 0.041 (− 0.6 to 0.5)	− 0.311 (− 1.0 to 0.3)	0.013 (− 3.3 to 3.4)	< 0.00001
Taiwan [Taipei]	− 0.136 (− 0.6 to 0.7)	0.0 (− 0.7 to 0.7)	− 0.517 (− 1.2 to − 0.3)	− 0.54 (− 0.8 to − 0.3)	− 0.447 (− 0.7 to − 0.3)	− 0.397 (− 0.7 to − 0.2)	− 0.494 (− 0.8 to − 0.3)	− 0.093 (− 1.5 to 1.2)	< 0.00001
Vancouver Island [Victoria]	− 0.227 (− 4.2 to 2.1)	0.0 (− 3.6 to 2.3)	− 0.285 (− 1.0 to 0.4)	− 0.028 (− 0.7 to 1.4)	0.321 (− 0.7 to 1.8)	− 0.504 (− 2.3 to 0.6)	0.147 (− 0.7 to 1.8)	− 0.139 (− 8.7 to 3.5)	0.3264
South Pacific Ocean									
Australia [Sydney]	− 0.118 (-0.7 to 0.3)	0.0 (− 0.5 to 0.4)	− 0.35 (− 0.7 to − 0.1)	− 0.525 (− 0.7 to − 0.3)	− 0.533 (− 0.8 to − 0.3)	− 0.364 (− 0.6 to − 0.1)	− 0.529 (− 0.8 to − 0.3)	− 0.09 (− 1.3 to 1.5)	< 0.00001
Australia—Tasmania [Hobart]	− 0.089 (− 1.0 to 0.9)	0.0 (− 0.7 to 1.0)	− 0.285 (− 0.7 to 0.0)	− 0.280 (− 0.4 to 0.0)	− 0.169 (− 0.3 to 0.0)	− 0.303 (− 0.5 to 0.1)	− 0.225 (− 0.4 to 0.0)	− 0.039 (− 1.2 to 1.5)	< 0.00001 (KW)
Indonesia—Borneo [Samarinda]	− 0.187 (− 1.2 to 0.9)	0.0 (− 0.8 to 0.8)	− 0.273 (− 0.5 to 0.0)	− 0.48 (− 0.7 to − 0.2)	− 0.49 (− 1.0 to − 0.1)	− 0.671 (− 1.1 to − 0.4)	− 0.485 (− 1.0 to − 0.1)	− 0.084 (− 1.4 to 2.0)	< 0.00001
Indonesia—Sulawesi [Makassar]	− 0.093 (− 0.5 to 0.2)	0.0 (− 0.2 to 0.3)	− 0.173 (− 0.3 to − 0.1)	− 0.243 (− 0.4 to 0.0)	− 0.279 (− 0.4 to − 0.1)	− 0.274 (− 0.4 to − 0.1)	− 0.261 (− 0.4 to 0.0)	− 0.033 (− 0.4 to 0.6)	< 0.00001
New Britain [Kimbe]	− 0.076 (− 0.3 to 0.1)	0.0 (− 0.2 to 0.2)	− 0.28 (− 0.4 to − 0.2)	− 0.287 (− 0.4 to − 0.2)	− 0.245 (− 0.3 to − 0.2)	− 0.21 (− 0.3 to − 0.1)	− 0.266 (− 0.4 to − 0.2)	− 0.02 (− 0.3 to 0.3)	< 0.00001
New Guinea [Jayapura]	− 0.126 (− 0.7 to 0.7)	0.0 (− 0.4 to 0.5)	− 0.083 (− 0.4 to 0.3)	− 0.133 (− 0.4 to 0.2)	− 0.266 (− 0.4 to − 0.1)	− 0.314 (− 0.5 to − 0.2)	− 0.199 (− 0.4 to 0.2)	− 0.057 (− 0.8 to 1.2)	< 0.00001
New Zealand—North Island [Auckland]	− 0.028 (− 0.6 to 0.7)	0.0 (− 0.5 to 0.5)	− 0.32 (− 0.5 to − 0.1)	− 0.295 (− 0.5 to 0.0)	− 0.206 (− 0.3 to 0.0)	0.022 (− 0.1 to 0.2)	− 0.251 (− 0.5 to 0.0)	0.007 (− 0.9 to 0.9)	< 0.00001
New Zealand—South Island [Christchurch]	− 0.058 (− 0.9 to 0.8)	0.0 (− 0.9 to 1.0)	− 0.393 (− 0.6 to − 0.1)	− 0.316 (− 0.7 to − 0.1)	− 0.355 (− 0.5 to 0.0)	− 0.171 (− 0.5 to 0.1)	− 0.335 (− 0.7 to 0.0)	− 0.067 (− 1.5 to 1.3)	< 0.00001
Indian Ocean									
Indonesia—Sumatra [Medan]	− 0.072 (− 0.6 to 0.5)	0.0 (− 0.5 to 0.5)	− 0.055 (− 0.5 to 0.4)	− 0.283 (− 0.6 to 0.0)	− 0.125 (− 0.5 to 0.4)	− 0.07 (− 0.5 to 0.1)	− 0.204 (− 0.6 to 0.4)	0.07 (− 0.8 to 1.3)	< 0.00001
Indonesia—Java [Jakarta]	− 0.057 (− 0.4 to 0.3)	0.0 (− 0.3 to 0.2)	− 0.082 (− 0.3 to 0.1)	− 0.119 (− 0.3 to 0.2)	− 0.182 (− 0.3 to 0.0)	− 0.16 (− 0.3 to 0.0)	− 0.15 (− 0.3 to 0.2)	− 0.01 (− 0.3 to 0.4)	< 0.0001 (KW)
Madagascar [Antananarivo]	− 0.171 (− 0.9 to 0.4)	0.0 (− 0.5 to 0.5)	− 0.361 (− 0.6 to − 0.1)	− 0.304 (− 0.5 to − 0.1)	− 0.368 (− 0.7 to − 0.2)	− 0.276 (− 0.5 to 0.1)	− 0.336 (− 0.7 to − 0.1)	− 0.023 (− 0.8 to 0.7)	< 0.00001
Sri Lanka [Colombo]	− 0.033 (− 0.5 to 0.4)	0.0 (− 0.4 to 0.5)	− 0.044 (− 0.3 to 0.2)	− 0.169 (− 0.5 to 0.2)	− 0.23 (− 0.5 to 0.0)	− 0.151 (− 0.4 to 0.0)	− 0.2 (− 0.5 to 0.2)	0.128 (− 0.9 to 1.9)	< 0.00001
Timor [Kupang]	− 0.066 (− 0.3 to 0.1)	0.0 (− 0.2 to 0.2)	− 0.167 (− 0.3 to 0.0)	− 0.158 (− 0.3 to 0.0)	− 0.157 (− 0.3 to 0.0)	− 0.14 (− 0.2 to − 0.1)	− 0.157 (− 0.3 to 0.0)	− 0.015 (− 0.3 to 0.3)	< 0.00001

*KW—Kruskal–Wallis test (used if the data were not normally distributed).

The year 1816 had the largest negative temperature anomalies and all 31 islands had these anomalies. They ranged from − 1.33 °C for Iceland to − 0.01 °C for Marajó. The next highest anomalies were seen for Great Britain (− 1.12 °C), and Honshū (− 0.91 °C). The subsequent year (1817) had the next largest negative anomalies after 1816 and these ranged from − 0.94 °C for Iceland to a positive value for Vancouver (0.32 °C). The next highest negative anomalies in 1817 were seen for Honshū (− 0.55), and Hispaniola (− 0.55).

The year 1818 had the smallest negative anomalies out of the four years (1815 to 1818) and 87% (27/31) of the islands had such negative anomalies. These were greatest for Borneo (− 0.67 °C), Iceland (− 0.56 °C), and Hispaniola (− 0.52 °C). There were no negative anomalies for the North Island of New Zealand, Marajó, Ireland and Great Britain (which had the highest positive anomaly at 0.69 °C). Overall, there was a small decline in anomalous temperatures of colder or equal to − 0.2 °C from 21 islands in 1816 to 17 islands in 1818. But complete returns to the reference period temperatures did not occur until the mid-1820s for both hemispheres (Fig. 2).

The statistical analysis comparing the months in the “non-volcanic” reference period (1779 to 1808) with the months in the peak Tambora impact years (1816–1817), typically found highly statistically significant differences (Table 4). The only islands where the differences were not significant were Great Britain, Marajó, Isla Grande de Tierra del Fuego, and Vancouver Island. The result for Great Britain was perhaps due to a mixed picture with colder temperatures in 1816 (highly significant for just that year, p = 0.0099), and less anomalous temperatures in 1817 (at − 0.09 °C).

Mean and median temperature anomalies in 1816 relative to the reference period are shown in Table 5. There were significantly larger anomalies (greater temperature reductions) for the islands in the Northern vs Southern Hemisphere (p < 0.0001). Larger anomalies were also seen for islands in the Atlantic Ocean and particularly the North Atlantic, compared to the Pacific and Indian Oceans (lowest in the latter). Similarly, islands in the tropics and subtropics had lower anomalies than those in the temperate zone (p < 0.0001), and more so if these were in the Southern vs Northern Hemisphere (i.e., Australia, Java, Madagascar, Marajó, New Britain, New Guinea and Timor; p = 0.0057).

**Table 5:** Mean and median temperature anomalies (°C) in 1816 relative to the “non-volcanic” reference period (1779 to 1808) using monthly data from the reconstruction EKF400v2 for all the islands in this study and analysed by location and relationship to reported food production and food insecurity.

Characteristic	Number of islands	Mean temperature anomaly (relative to reference period)	SD	Median temperature anomaly (relative to reference period)	p-value
Hemispheres*					
Northern Hemisphere (NH)	17	− 0.49	0.51	− 0.40	< 0.0001 (KW)
Southern Hemisphere (SH)	11	− 0.23	0.23	− 0.26	
Main oceans spanning both hemispheres					
Atlantic Ocean**	9	− 0.52	0.61	− 0.43	< 0.0001 (KW)
Pacific Ocean	17	− 0.36	0.31	− 0.34	
Indian Ocean***	5	− 0.21	0.16	− 0.19	
Oceans by hemisphere*					
North Atlantic Ocean**	7	− 0.65	0.61	− 0.52	< 0.0001 (KW)
North Pacific Ocean	9	− 0.39	0.39	− 0.36	
North Indian Ocean	1	− 0.17	0.20	− 0.10	
South Atlantic Ocean	2	− 0.05	0.34	− 0.06	
South Pacific Ocean	6	− 0.31	0.18	− 0.32	
South Indian Ocean	3	− 0.19	0.13	− 0.19	
Tropics and subtropics vs temperate zones^#^					
Temperate zone	13	− 0.51	0.55	− 0.39	< 0.0001 (KW)
Tropics and subtropics	18	− 0.29	0.25	− 0.27	
Temperate zones by hemisphere					
NH temperate zone	9	− 0.63	0.61	− 0.53	< 0.0001 (KW)
SH temperate zone	4	− 0.25	0.23	− 0.28	
Tropics and subtropics by hemisphere					
NH tropics and subtropics	8	− 0.33	0.27	− 0.29	0.0057 (ANOVA)
SH tropics and subtropics	7	− 0.22	0.23	− 0.25	
Food production impaired^##^					
Reported	8	− 0.55	0.61	− 0.37	0.0167 (KW)
Nil reported or unknown	23	− 0.32	0.31	− 0.30	
Food insecurity^##^					
Reported	4	− 0.84	0.73	− 0.83	< 0.0001 (KW)
Nil reported, unknown, or not definitive	27	− 0.31	0.30	− 0.29	

*KW* Kruskal–Wallis test (if the data were not normally distributed), *SD* Standard deviation.

*Excluding the three islands that straddle the equator: Borneo, Sulawesi and Sumatra.

**The Atlantic and North Atlantic groupings included islands in the Caribbean (Cuba and Hispaniola) and in the Mediterranean (Sicily).

***Including Java, Madagascar, Sri Lanka, Sumatra, and Timor. Although Australia borders both the Indian and Pacific Oceans, we classified it as in the South Pacific for this analysis.

^#^As per this map of the tropics, subtropics and temperate zones: https://commons.wikimedia.org/wiki/File:World_map_indicating_tropics_and_subtropics.png (that is, the following islands were included in the subtropical zone: Taiwan, the Japanese island of Kyūshū, and Australia (main continental island which is mainly in the tropics and subtropics, with a smaller southern part in the temperate zone)).

^##^See Table 1 (for definite evidence only and excluding the “probably yes” countries).

There was also some relationship between the reconstruction findings and those from the literature review (Table 1). That is for islands reporting impaired food production or food insecurity, there were greater temperature anomalies (greater reductions) than the other islands (p = 0.0167 and p < 0.0001 respectively).

## Discussion

### Main findings and interpretation

The reconstruction data indicates that the islands in this study had lower temperature anomalies in the 1815 to 1818 period when compared to latitudinally equivalent sites (at 100 km and 1000 km inland) on the nearest continent. Such patterns likely reflect the well-known heat store and thermal moderating capacity of the oceans. Furthermore, the previous descriptions of famines associated with the Mt Tambora eruption were particularly in continental regions—i.e., Western and Central Europe, India and China^21^. In terms of the peak temperature reduction for the islands in the Northern Hemisphere (− 0.49 in 1816, Table 5), this was around half the median impact estimated for global land areas at the peak of the climate impact. The latter was from eight different studies of the Tambora eruption for peak impacts (with a median of − 0.975 °C; ranging from − 0.875 to − 1.3)^35^. The pattern of lower temperature after Tambora was also consistent with other work that utilised a different reference period for comparison (i.e., 1851 to 1900, as per Fig. S6 in Reichen et al.^36^ albeit considering both Tambora and a circa 1809 eruption together). This work by Reichen et al. also indicated lower temperature impacts on islands in the Northern Hemisphere relative to continental land masses.

Both the island-specific evidence identified in the literature review and the analysis of reconstruction data indicate that nearly all these 31 islands had anomalous temperature reductions in at least one of the years following the Tambora eruption. This is not surprising given the other published evidence relating to the widespread impacts of this particularly large magnitude 7 eruption (see “Introduction” section). Furthermore, there was a statistically significant relationship between the literature review and the reconstruction findings. That is for islands reporting impaired food production or food insecurity, there were greater temperature anomalies (greater reductions) than the other islands. Nevertheless, the island-specific evidence from the literature review remains far from complete and further historical and palaeoclimate research is desirable to provide a more comprehensive picture.

The analysis of the reconstruction data indicated less anomalous temperature impacts for islands in the Southern Hemisphere compared to the Northern Hemisphere. This hemispheric pattern has been reported for other studies of the Tambora eruption^20,37^. Similarly, our findings for islands are similar to other work that has reported relatively greater temperature impacts of this eruption in the North Atlantic region (Western Europe and Eastern North America^21^) and for the Northern Hemisphere extratropics compared to the Southern Hemisphere extratropics^21^.

The stronger cooling seen in the Northern Hemisphere after the eruption, probably reflects larger cooling over land than oceans^20^. Indeed, this is despite ice core data^38^ and modelling work^39^, suggesting that aerosols ejected into the stratosphere from the Tambora eruption were at higher levels in the Southern Hemisphere than the Northern Hemisphere (Mt Tambora is located just south of the equator at latitude 8 degrees south).

While the Tambora eruption is relatively good to study because of its large magnitude (e.g., when compared to the temperature reduction impact of seven other eruptions during the last phase of the Little Ice Age^33^), it has the complexity of potentially being part of a multi-eruption impact. That is, ‘part of this cooling might have been due to a previous “unknown” eruption (a volcanic layer documented in ice cores, which could not yet be attributed to a known eruption) circa 1809’^21^ (see also Timmreck et al.^40^). Some impact from this unknown eruption was however included in the EKF400v2 reconstruction (which used volcanic forcing data from Crowley et al.^41^) and this may explain the temperature decline in the Northern Hemisphere in 1810 shown in Fig. 2.

### Study strengths and limitations

A strength of this study is that it is the first (that we are aware of) to specifically explore the impact of a major volcanic eruption on a set of large, populated islands. Also, we were able to collate a wide range of literature—with some island-specific impacts being described by many different studies using different data sources (see Table S2 in the Supplementary Information File 1). We were also able to use the results of a recent reconstruction: EKF400v2 that has previously been found to perform well in describing a major historical European drought and ENSO effects in the nineteenth century (see “Methods” section). Nevertheless, our study still has many limitations, as summarised below:

- *Gaps in data from the literature review* In the review work there were frequent information gaps, particularly on whether or not food production or food insecurity were impacted (Fig. 1, Table 1). This partly reflects those islands where the indigenous population did not have written records or if any colonial authorities on the island did not keep such records. An example of the latter was New Zealand vs Tasmania (islands on similar latitudes), where both had palaeoclimate evidence for Tambora impacts on weather/climate, but only Tasmania had recorded impacts on crop production. In contrast, the European population on New Zealand in 1815–1817 was very small and probably largely illiterate. But in other cases, written records may exist but historians have not yet documented these in relationship to the Tambora eruption (e.g., for places with possible unpublished written records on food prices such as Sicily). There may also have been palaeoclimate studies missed in our literature searches as some such studies cover multiple volcanic forcings but do not always include the word “Tambora” anywhere in the text.
- *Food insecurity can reflect more than climate impacts* While we identified some apparent food insecurity impacts (Table 1), it is important to note that famines can be substantially socioeconomic phenomena as shown by Amartya Sen^42^. That is, famines can reflect the extent to which people have money to pay for food and if food is redistributed by authorities to the needy (as indeed occurred in parts of Europe in 1817 in response to the Tambora impacts^43^). Also, the extent of food trade within the island and from outside the island can be relevant. For example, internal trade in rice may have somewhat buffered various parts of Japan in some historical famine periods^44^ and there was trade in rice between Indonesian islands in 1815 (e.g., between Bali and Java^45^). Madagascar also exported rice to Africa and the Mascarene Islands at this time—but the country still suffered regular famines, with roles in some of these famines played by epidemics (e.g., of smallpox) and internal conflict such as raids for slaves and cattle^46^. Food insecurity may also be avoided if a locality had pre-existing over-production capacity or was able to divert crops to feed humans away from other uses (e.g., as animal feed or for brewing alcoholic beverages). Malnutrition can also be disguised, and this was possibly the case for Sicily in 1817. It did not suffer “famine”, but had a typhus epidemic in this year^47^. However, it is possible that this typhus epidemic was facilitated by poor nutrition associated with poorer harvests—as was the case in Ireland (see Table S2, Supplementary Information File 1).
- *Limits with palaeoclimate data* Both the literature review findings and the reconstruction (EKF400v2) were partly informed by regional and island-specific palaeoclimate data (Table S1 in Supplementary Information File 1). While such data are increasingly incorporated into climate models by climate scientists, there are still relevant limitations. For example, data from tree-ring studies may over-estimate temperature impacts from volcanic eruptions (since tree growth is also lowered by reduced light)—at least at high-latitudes^48^. On the other hand, tree-ring studies from moisture-stressed sites may fail to capture extreme low temperature events from volcanic eruptions^49^.
- *Other impacts on climate* In addition to the mystery circa 1809 eruption at an unknown site (see above), there was also a period of low solar activity known as the Dalton Minimum from 1790 to 1830^21^. The ENSO has also been suggested as potentially contributing to some of the cooling after Tambora in 1817 for the “Indonesian Warm Pool Region”^50^. Although the EKF400v2 reconstruction that we used does assimilate ENSO effects, it may still not do this optimally for all of the included islands. There may also have been an impact of the “North Atlantic Oscillation” pattern, but one study observed no impact from it on the modelled Tambora effect^20^.
- *Other limits with volcanic eruption reconstructions* While reconstructions such as EKF400v2 can be validated against historical events (droughts and ENSO effects—see “Methods” section) there are still limitations. For example, there is variation in models of the Tambora eruption in estimating stratospheric global mean aerosol optical depth (e.g., reflecting considerations of hydroxyl radical chemistry following volcanic injection of sulphur dioxide)^51^. Also in our analysis we focused on just the temperature impacts from the EKF400v2 reconstruction (for the reasons detailed in the “Methods” section) and yet there is evidence that volcanic eruptions can reduce precipitation in wet tropical regions (from both observational data and modelling^52^), and decreased monsoon rainfall^33,52^. The grid cell size used in the EKF400v2 reconstruction is still relatively large at two degrees of latitude and longitude square (i.e., around 222 km^2^ at the equator). Also, the grid cells are dichotomised into either land or sea, thereby simplifying detail for coastal areas that have peninsulas etc. Finally, the statistical analyses relating to the reconstruction data need to be interpreted with some caution given that some of the “raw data” (that is combined with the atmospheric modelling) is a mix of reconstructed values from palaeoclimate data etc., and interpolated values for localities with no such data.

### Potential implications for research and policy

Given the uncertainties and study limitations detailed above, there is a need for additional research on the impact of the Tambora eruption and other historical large magnitude volcanic eruptions. Ideally this should involve additional paleoclimate data (e.g., from tree-rings, coral samples etc.) and historical weather data, and be integrated with state-of-the-art climate simulations. The ideal such reconstructions should both assimilate observational data (as per EKF400v2) but also include climatic impacts on different types of crops (as per Kandlbauer et al.^20^). Complex impacts on sea-ice and oceans of reduced sunlight also need to be considered (including impacts on fisheries), as per work on nuclear winter^9^. There are also qualitative differences between nuclear winter and volcanic winters that could be considered (e.g., differing: time periods of aerosols in the stratosphere, levels of acid rain, damage to the ozone layer and the radionuclides from nuclear war). Further work with historical records on food prices in markets may also clarify food production and food insecurity issues in some islands after the Tambora eruption.

The relevance of the current results to the selection of potential island refuges for humanity to best survive sunlight-reducing catastrophes should still be considered provisional. Nevertheless, the findings do point to the likely benefits of island refuges in the Southern Hemisphere, the Indian Ocean and the tropics and subtropics of the Southern Hemisphere. But other considerations for island refuge location are the findings of simulation studies of the global climate impacts of nuclear war (see “Introduction” section), and the risk of islands being directly attacked in a nuclear war (e.g., those in military alliances with nuclear weapon states such as Australia, Iceland and Japan). Other relevant features of island refuges include excess food production capacity^8,53^, capacity to survive extreme pandemics^18^, and to have the socio-economic and technological characteristics to be a “node of persisting complexity”^17^.

## Conclusions

The findings of both the literature review and reconstruction simulations suggest climatic impacts of the Tambora eruption for nearly all these 31 large islands. These were smaller impacts than for latitudinally equivalent continental sites. Islands with the smallest temperature anomalies were in the Southern Hemisphere, in particular the Indian Ocean and the tropics and subtropics of the Southern Hemisphere. This does provide some information for the selection of potential island refuges for humanity to best survive sunlight-reducing catastrophes, but many other factors need to be considered. There also remain many gaps in the historical record of the impact of the Tambora eruption and other limitations persist with the reconstruction data.

## Supplementary Information

Supplementary Legends.

Supplementary Information 1.

Supplementary Information 2.

Supplementary Information 3.

## Data Availability

The data are all are contained in the manuscript and in the three Supplementary Information files.
