# Supplementary material for: Impact of the Tambora volcanic eruption of 1815 on islands and relevance to future sunlight-blocking catastrophes
Source: Sci Rep. 2023 Mar 4;13:3649. doi: 10.1038/s41598-023-30729-2 (PMC9985606; doi:10.1038/s41598-023-30729-2)
Supplement: Supplementary file 1 — Supplementary Legends. [file 41598_2023_30729_MOESM1_ESM.docx]

**Supplementary Information File 1 (Table and Figure legends)**

Table S1: Island-specific data relating to the time of the Tambora eruption assimilated into the EKF400v2 reconstruction (for specific references see Valler et al^1^)

Table S2: Identified information on the potential weather/climate and food impacts of the Tambora eruption of 1815 on islands for any of the years 1815-17 (for the 31 selected islands – see *Methods* in main manuscript; with assessments of an overall impact of “probably yes” or “probably no” where the evidence was mixed)

Figure S1: The latitudinally equivalent continental sites for each of the selected islands, used for the Tambora impact comparisons between islands and continents (Image produced using Ferret v7.63)
