# Supplementary material for: Impact of the Tambora volcanic eruption of 1815 on islands and relevance to future sunlight-blocking catastrophes
Source: Sci Rep. 2023 Mar 4;13:3649. doi: 10.1038/s41598-023-30729-2 (PMC9985606; doi:10.1038/s41598-023-30729-2)
Supplement: Supplementary file 2 — Supplementary Information 1. [file 41598_2023_30729_MOESM2_ESM.pdf]

Supplementary Information

Supplementary Information for Wilson et al "Impact of the Tambora Volcanic Eruption of 1815 on Islands and Relevance to Future Sunlight-Blocking Catastrophes"

Table S1: Island-specific data relating to the time of the Tambora eruption assimilated into the EKF400v2 reconstruction (for specific references see Valler et al[1])

**Island in this study** (see Table 1 in the main manuscript for details on jurisdictional status in the 1815-1817 period and currently)	**Assimilated data**
North Atlantic Ocean (includes Caribbean and Mediterranean)	
Cuba	None
Great Britain	Temperature, precipitation, sea-level pressure (SLP), pressure, wet days, tree-ring
Hispaniola	None
Iceland	None
Ireland	Temperature, precipitation, SLP, pressure, wet days, tree-ring
Newfoundland	None
Sicily	Temperature, precipitation, SLP, pressure, wet days, tree-ring
South Atlantic Ocean	
Isla Grande de Tierra del Fuego	Tree-ring
Marajó (Brazil)	None
North Pacific Ocean	
Hainan	None
Japan – Honshū	Documentary record, tree-ring
Japan – Hokkaidō	Tree-ring
Japan – Kyūshū	Tree-ring
Philippines – Luzon	None
Philippines – Mindanao	None
Sakhalin	Tree-ring
Taiwan	Tree-ring
Vancouver Island	Tree-ring
South Pacific Ocean	
Australia (main island continent)	Tree-ring
Australia – Tasmania	Tree-ring
Indonesia – Borneo	None
Indonesia – Sulawesi	Tree-ring
New Britain	None
New Guinea	None
New Zealand – North Island	Tree-ring
New Zealand – South Island	Tree-ring
Indian Ocean	
Indonesia – Sumatra	None
Indonesia – Java	Tree-ring, coral
Madagascar	None
Sri Lanka	None
Timor	None

Figure S1: The latitudinally equivalent continental sites for each of the selected islands, used for the Tambora eruption impact comparisons between islands and continents (Image produced using Ferret v7.63)

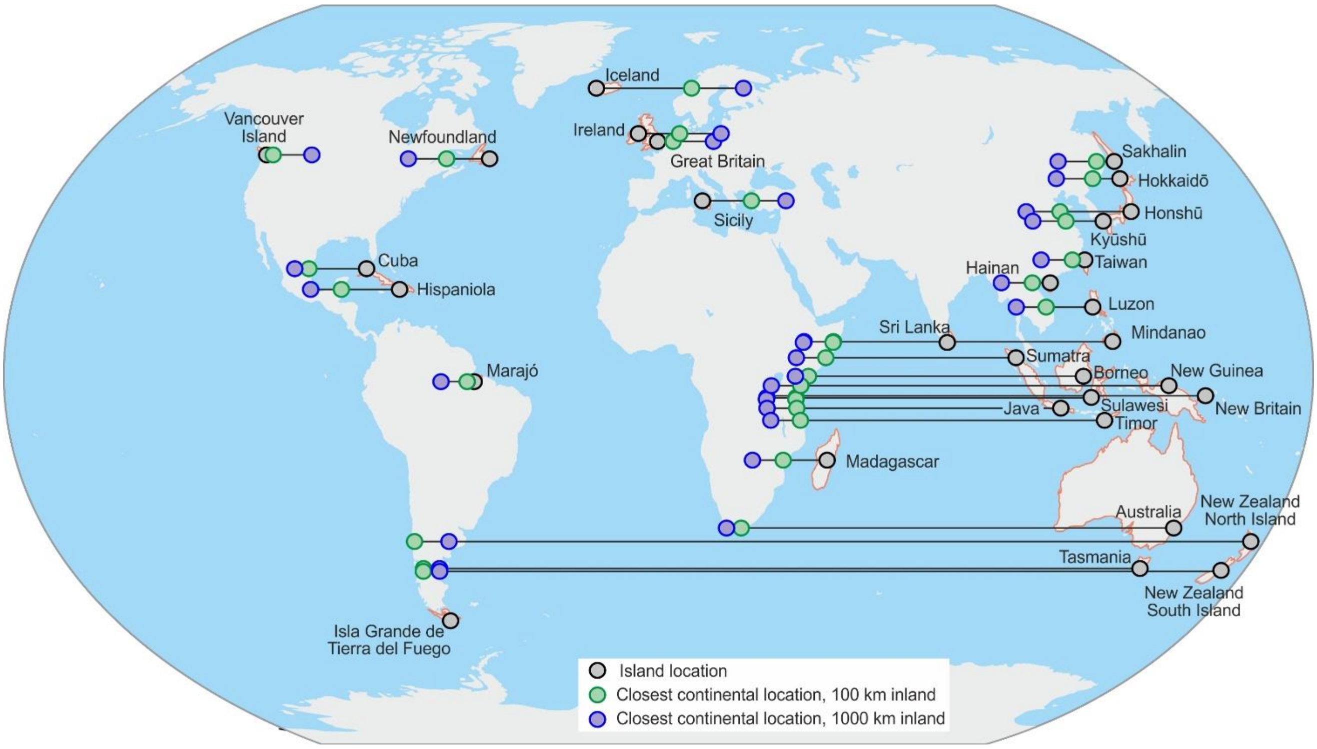

Table S2: Identified information on the potential weather/climate and food impacts of the Tambora eruption of 1815 on islands for any of the years 1815-17 (for the 31 selected islands – see *Methods* in main manuscript; with assessments of an overall impact of "probably yes" or "probably no" where the evidence was mixed)

Island (as in 1815-17)	Anomal-ous weather/ climate	Adverse impacts on food production	Food insecurity	Comments / sources
North Atlantic Ocean (includes Caribbean and Mediterranean)				
Cuba (then part of the Spanish Empire)	Yes [Zhang]	Yes [Zhang]	–	Crop failures were attributed to unusually cold weather associated with the Tambora eruption (Zhang et al[2]). ***Regional context:*** These problems were also reported in another part of the Caribbean: ie, the British West Indies[2]. Similarly for the Bahamas (with Nassau being ~300 km away) there was instrumental data for cooling after Tambora (maximum cooling estimated at 1.0 °C)[3]. The 1810s were also a period of extreme drought in the Bahamas.
Great Britain (England, Scotland, Wales)	Yes [Briffa] [Wilson] [Dawson] Harington] [Brönnimann] [Lee] [Veale] [Schurer] [Brugnara] [Raible] [Reichen]	Yes [Harington] [Lee] [Veale]	Yes [Post] [Behringer] [Brönnimann]	A study using instrumental data for central England suggested a cooling impact after Tambora[4]. There was also an increase in gale-days in Scotland[5]. Archival data also support extremes of cold and wet weather at this time[6]. Reduced harvests (quantity and quality) were also reported[6]. There were increased food prices, and "fragmentary data point to a noticeable rise in death rates"[7]. A typhus epidemic in Scotland and England was also probably partly related to the food insufficiency[7], and food-related riots also occurred[8 9]. Further details are in many sources eg, Harington[10], Behringer[8], Brönnimann and Krämer[9], and Lee and MacKenzie[11]. ***Regional analyses:*** In a regional analysis that included instrumental data (from 4 stations in England and Scotland) and regional tree-ring data, there was evidence of temperature reductions and precipitation anomalies in 1816[12] (see also earlier work by some of the same authors[13]). Another regional analysis using instrumental data, proxies and modelling, reported increased precipitation (Fig. 9 [left panel] in: [9]). In a regional analysis using tree-ring data and modelling, there was a higher Palmer Drought Severity Index (PDSI) score (lower drought risk) (Fig. 9 [right panel] in: [9]). Another regional analysis using instrumental data, proxies and modelling, reported temperature reductions and precipitation increases (Fig. 10 in: [9]). Another regional analysis using observation data from weather stations and ship logs, reported temperature reductions and increased precipitation (Fig. 2 in: [14]).

Island (as in 1815-17)	Anomal-ous weather/ climate	Adverse impacts on food production	Food insecurity	Comments / sources
				Another regional analysis using instrumentation data from weather stations and ship logs (including data from Northern Ireland) found anomalous circulation/air pressure patterns[15]. Another regional analysis using weather station data (from Casty et al[16]) and modelling, reported temperature reductions and increased precipitation[17]. Another regional analysis using phenological data (freezing and thawing dates of rivers, plant observations) indicated colder temperatures (albeit considering both Tambora and a circa 1809 eruption together, as per Fig. S6 in: [18]).
Hispaniola (now Haiti and Dominican Republic)	–	–	–	No specific data identified. ***Regional context:*** Data from nearby Cuba (~90km away) and Bahamas (~700 km away) provides some suggestion of a regional climatic impact (see the details for Cuba above).
Iceland (then part of the Danish Empire)	Yes [Ogilvie] [Brönnimann] [Schöne] [Schurer] [Raible]	Yes [Ogilvie]	Yes [Ogilvie]	Increased ice impeded fishing and vegetable crops (potatoes, cabbage and turnips) suffered[19]. Unusual sea ice conditions around Iceland have also been detailed elsewhere (Fig. 27 in: [9]). Data from a bivalve mollusc study also support a temperature reduction[20]. The severity of the impacts on food production was indicated by begging being reported, albeit this was limited to one district[19]. ***Regional analyses:*** In regional analyses using instrumental data, proxies and modelling, there were temperature reductions (Fig. 9 and Fig. 10 in: [9]). But results for precipitation varied by data source and model (ie, reductions in Fig. 9 and increases in Fig. 10). Another regional analysis using observation data from weather stations and ship logs, reported temperature reductions and also reduced precipitation (Fig. 2 in[14]). Another regional analysis using weather station data (from Casty et al[16]) and modelling, reported temperature reductions and reduced precipitation[17].
Ireland (then in a union with Great Britain)	Yes [Wilson, 1999] [Murphy] [Briffa] [Harington] [Brönnimann] [Schurer] [Brugnara] [Raible] [Reichen]	Yes [Wilson, 1992]	Yes [Post] [Behringer] [Brönnimann]	Instrumental data suggest a cooling after Tambora (data collected in Armagh)[4]. A 305-year continuous monthly rainfall series found that the summer 1817 was the fourth wettest, but the summer of 1816 was only the 53rd wettest[21]. There was damage to crops[22], increased food prices[8] and food-related riots[9]. As well as an increase in begging and famine[7], there were deaths from a major typhus epidemic[8], and an epidemic of smallpox[7]. Malnutrition probably contributed to these epidemics (with this also being a view concerning typhus of an Irish physician at the time[7]). Further details are in many sources eg, Harington[10], Behringer[8], and Brönnimann and Krämer[9]. ***Regional analyses:*** In a regional analysis that included instrumental data (from neighbouring England and Scotland) and regional tree-ring data, there were estimates of

Island (as in 1815-17)	Anomal-ous weather/ climate	Adverse impacts on food production	Food insecurity	Comments / sources
				temperature reductions and precipitation anomalies in 1816 for Ireland[12] (see also earlier work by some of the same authors[13]).<br>Another regional analysis using instrumental data, proxies and modelling, there was increased precipitation (Fig. 9 [left panel] in: [9]).<br>Another regional analysis using tree-ring data and modelling, reported a higher PDSI score (lower drought risk) (Fig. 9 [right panel] in: [9]).<br>Another regional analysis using instrumental data, proxies and modelling, there were temperature reductions and precipitation increases (Fig. 10 in: [9]).<br>Another regional analysis using observation data from weather stations and ship logs, reported temperature reductions but no change in precipitation (Fig. 2 in: [14]).<br>Another regional analysis using instrumentation data from weather stations and ship logs (including data from Northern Ireland) found anomalous circulation/air pressure patterns[15].<br>Another regional analysis using weather station data (from Casty et al[16]) and modelling, reported temperature reductions and increased precipitation[17].<br>Another regional analysis using phenological data (freezing and thawing dates of rivers, plant observations) indicated colder temperatures (albeit considering both Tambora and a circa 1809 eruption together, as per Fig. S6 in: [18]).
Newfoundland (then part of the British Empire, now part of Canada)	Yes<br>[Brönnimann]<br>[Devor]<br>[Newell]	Yes<br>[American Advocate]	Yes<br>[American Advocate]	There was also evidence of increased sea ice around Newfoundland at this time[23]. Newell reported that the summer 1816 conditions were similar to the current day March and April (spring) conditions[24]. Newell also reported that catch statistics for cod in 1816 in Newfoundland waters suggested “a relatively good year”, although the weather was not favourable for drying the cod[24]. But in the following year, 1817, a media report refers to “failure of the harvest” and a “starving populace”, with a violent mob attacking a vessel (presumably for food)[25].<br>***Regional analyses:*** In a regional analysis using instrumental data, proxies and modelling, there were temperature reductions and precipitation increases (Fig. 10 in: [9]).<br>In another regional analysis using tree-ring data and modelling, there was a higher PDSI score indicating lower drought risk (Fig. 9 in: [9]).<br>A regional analysis using tree-ring data suggested reduced temperature for Newfoundland[13].<br>***Regional context:*** The conditions in neighbouring Labrador (~20 km away) also involved reports of hunger in the Inuit[26]. Famine was also described in parts of Eastern Canada (north-western Ontario)[27].

Island (as in 1815-17)	Anomal-ous weather/ climate	Adverse impacts on food production	Food insecurity	Comments / sources
Sicily (then part of a kingdom of Southern Italy)	Yes [Briffa] [Brönnimann] [Raible]	–	Probably yes (given a typhus epidemic) [Post]	It was reported that “although famine spared Sicily, the island was not exempt from typhus” in 1817 (p128 in: [7]). Nevertheless, this epidemic probably reflected some degree of poorer nutrition in the population related to anomalous weather (eg, given the links with typhus and poorer nutrition in Ireland after Tambora). However, additional literature searches on this particular epidemic did not clarify this issue.<br>***Regional analyses:*** In a regional analysis that included instrumental data (including from Palermo in Sicily) and regional tree-ring data, there was evidence of temperature reductions in Sicily[12] (see also earlier work by some of the same authors[13].)<br>In another regional analysis using instrumental data, proxies and modelling, there were temperature reductions but no change in precipitation (Fig. 10 in: [9]). In another regional analysis using tree-ring data and modelling, there was a lower PDSI score reflecting increased drought risk (Fig. 9 [right panel] in: [9]).<br>Another regional analysis using weather station data (from Casty et al[16]) and modelling, reported temperature reductions and reduced precipitation[17].<br>***Regional context:*** There are also tree-ring data suggesting small temperature reductions for another island in the Mediterranean, Corsica. These changes were less than seen for the Alps and Pyrenees in Europe at this time (Fig. 6 in Szymczak et al[28]).
South Atlantic Ocean				
Isla Grande de Tierra del Fuego (now part of both modern day Chile and Argentina)	Yes [Villalba]	–	–	Tree-ring data from 4 species (and a composite measure) indicate a statistically significant three year cooling[29]. But the authors suggested that the impact may have been reduced by the El Niño-Southern Oscillation (ENSO) effect.
Marajó (Brazil) (then part of the Portuguese Empire)	–	–	–	No specific data identified. This island is in the mouth of the Amazon River.
North Pacific Ocean				
Hainan (an island province of China)	Yes [Gao] [Wilson]	Yes [Wilson]	No [Gao]	Unusual snow falls were reported in Hainan[30] and rice was killed by frost[22]. However, out of all Chinese provinces, famine and increased mortality were only reported in the south-western province of Yunnan (in 29 of the 88 counties)[30].<br>The estimated temperature anomaly (from tree-ring data and historical records) was also less than most other locations in China (see Fig. 2 in: [30]).<br>***Regional analysis:*** In a regional analysis using instrumental data, proxies and modelling, there was increased precipitation (Fig. 9 [left panel] in: [9]).

Island (as in 1815-17)	Anomal-ous weather/ climate	Adverse impacts on food production	Food insecurity	Comments / sources
				A Western Pacific Ocean wide analysis from coral samples (albeit with no Hainan samples), reported sea surface temperature reductions after the Tambora eruption (albeit with some likely influence from an unknown eruption in 1808/1809)[31].
Japan – Honshū	Probably yes overall:<br><br>Yes:<br>[Mikami & Tsukamura]<br>[Brönnimann]<br>[Ohyama]<br>[Mikami, 2008]<br>[Tsukamura]<br>[Reichen]<br><br>No:<br>[Aono] [Ano and Kazui]	No<br>[Bassino]<br>[Tsukamura]	No<br>[Saito]	Tree-ring data for central Honshū is suggestive of a cooling from Tambora, but not for north-east Honshū[32]. Tsukamura reported no evidence of cool weather for the islands of Japan (including data for Honshū), based on historical records, though the mid-summer season came earlier in 1816 and persisted for longer[33]. This author also reported no crop losses in Japan for 1816.<br>Another study reported the summer of 1816 as being hot and dry, and that the winter of 1815-16 was more severe in central Japan[34]. A review included some data suggestive of cooling at the time of the Tambora eruption in graphs of lake freezing, tree-ring data, and historical weather records (Figs. 4, 6, 7, 10b)[35]. But for reconstructed spring temperatures in Kyoto based on cherry blossom records, there was no significant change in this period (the only cold period in this analysis in the 1800s was 1825–1830)[36]. A similar lack of impact was from reconstructed temperature from cherry blossom data for Edo (Tokyo)[37]. Reconstructed temperature for Tokyo from weather records in diaries could be consistent with a small Tambora impact, but the picture was not clear (Fig. 3 in Mikami 1996[38]).<br>Data on rice prices showed no unusual patterns (13 of the 15 markets with price data were on Honshū)[39]. Also, no famines in Japan were identified in the 1804 to 1822 period in a study that identified 136 countrywide and 98 regional famines[40].<br>***Regional analysis:*** In a regional analysis using tree-ring data (including from neighbouring Hokkaidō[41]) and modelling, there was generally a higher PDSI score (lower drought risk) (Fig. 9 in: [9]). The same results (using the same source data) are also presented elsewhere (Fig. 1 in: [42]).<br>Another regional analysis using phenological data (freezing and thawing dates of rivers, plant observations) indicated colder temperatures (albeit considering both Tambora and a circa 1809 eruption together, as per Fig. S6 in: [18]).
Japan – Hokkaidō	Probably yes overall:<br>Yes<br>[Brönnimann]<br>[Reichen]<br>Mixed:	No<br>[Tsukamura]	Probably no<br>[Saito]	Tsukamura reported no evidence of cool weather for the islands of Japan (including data for Hokkaidō), based on historical records, though the mid-summer season came earlier in 1816 and persisted for longer[33]. This author also reported no crop losses in Japan for 1816. While no famines in Japan were identified in the 1804 to 1822 period (see as per Honshū), the relevant dataset only included part of Hokkaidō in this period (the part in Tokugawa territory)[40].

Island (as in 1815-17)	Anomal-ous weather/ climate	Adverse impacts on food production	Food insecurity	Comments / sources
	[Tsukamura]			***Regional analysis:*** In a regional analysis using tree-ring data (including from Hokkaidō[41]) and modelling, there was a higher PDSI score (lower drought risk) (Fig. 9 in: [9]) (see as per Honshū).<br>Another regional analysis using phenological data (freezing and thawing dates of rivers, plant observations) indicated colder temperatures (albeit considering both Tambora and a circa 1809 eruption together, as per Fig. S6 in: [18]).
Japan – Kyūshū	Yes<br>[Tsukamura]	No<br>[Bassino]<br>[Tsukamura]	No<br>[Saito]	Tsukamura reported that the mid-summer season came earlier in 1816 and persisted for longer for the islands of Japan – with historical data also covering Kyūshū (see as per Honshū)[33]. This author also reported no crop losses in Japan for 1816.<br>Data on rice prices showed no unusual patterns (albeit the data for two markets in Kyūshū were combined with data for 13 markets on Honshū)[39]. Also, no famines in Japan were identified in the 1804 to 1822 period (see as per Honshū)[40].
Philippines – Luzon (then part of the Spanish Empire)	Yes<br>[Inoue]<br>[Brönnimann]<br>[Tierney]	–	Probably no<br>[Warren]	A study of coral off the coast of Luzon found that sea surface temperature “cooled relatively sharply by ~2.0 °C just after the Tambora eruption, which was associated with a substantial drought…”[43]. The drought effects were based on sea surface salinity estimates.<br>A study of food shortages and famines in the Philippines since the 1600s did not identify any such events in the 1815-17 period, but there was no detailed listing of all events[44].<br>***Regional analyses:*** In a regional analysis using instrumental data, proxies and modelling, there was decreased precipitation (Fig. 9 [left panel] in: [9]).<br>In another regional analysis using tree-ring data (including from the Philippines[41]) and modelling, there was a higher PDSI score (lower drought risk) (Fig. 9 [right panel] in: [9]).<br>A Western Pacific Ocean wide analysis from coral samples (albeit with no samples from the Philippines) reported sea surface temperature reductions after the Tambora eruption[31].
Philippines – Mindanao	Yes<br>[Brönnimann]<br>[Tierney]	–	Probably no<br>[Warren]	A study of food shortages and famines in the Philippines since the 1600s did not identify any such events in the 1815-17 period (see as per Luzon)[44].<br>***Regional analyses:*** In a regional analysis using instrumental data, proxies and modelling, there was decreased precipitation (Fig. 9 [left panel] in: [9]).<br>A Western Pacific Ocean wide analysis from coral samples (albeit with no samples from the Philippines) reported sea surface temperature reductions after the Tambora eruption (see as per Luzon)[31].
Sakhalin (part of modern day Russia and north of Japan. In 1815-17 it was	Yes<br>[Brönnimann]<br>[Reichen]	–	–	***Regional analysis:*** In a regional analysis using tree-ring data (including from Sakhalin[41]) and modelling, there was a higher PDSI score (lower drought risk) (Fig. 9 in: [9]). The same results are also presented elsewhere (Fig. 1 in: [42]).

Island (as in 1815-17)	Anomal-ous weather/ climate	Adverse impacts on food production	Food insecurity	Comments / sources
under no particular colonial rule).				Another regional analysis using phenological data (freezing and thawing dates of rivers, plant observations) indicated colder temperatures (albeit considering both Tambora and a circa 1809 eruption together, as per Fig. S6 in: [18]).
Taiwan (a province of China in 1815-17)	Yes [Gao] [Ramos] [Brönnimann] [Tierney]	–	No [Gao]	Unusual snow falls were reported in Taiwan, but no famine or increased mortality[30]. The estimated temperature anomaly (from tree-ring data and historical records) was also less than other locations in China (see Fig. 2 in: [30]). Analysis of a coral sample from the coast of Taiwan was suggestive of a small sea surface temperature change following 1815 (see Fig. 3a in: [45]), but not in sea surface salinity (Fig. 7a in: [45]). ***Regional analyses:*** Decreased precipitation was reported in a regional analysis using instrumental data, proxies and modelling (Fig. 9 [left panel] in: [9]). A Western Pacific Ocean wide analysis from coral samples (albeit with no Taiwan samples) reported sea surface temperature reductions after the Tambora eruption[31].
Vancouver Island (then part of the British Empire, now Canada). This island was populated with Indigenous peoples in 1815-17 and there was no European settlement.	Probably yes overall: Yes [Briffa] [Reichen] No: [Parker] [Brönnimann]	–	–	Parker reported no evidence of a climate impact based on tree-ring data from this island[46]. ***Regional analysis:*** In a regional analysis using tree-ring data and modelling, there was no change in the PDSI score (for drought risk) (Fig. 9 in: [9]). In contrast, another regional analysis using tree-ring data suggested reduced temperature for Vancouver Island[13]. Another regional analysis using phenological data (freezing and thawing dates of rivers, plant observations) indicated colder temperatures (albeit considering both Tambora and a circa 1809 eruption together, as per Fig. S6 in: [18]). ***Regional context:*** A study of bivalve mollusc samples suggested "conspicuously cool conditions in 1817 and 1818" for islands that are north of Vancouver (Tree Nob Islands, ~420km away)[47]. But this study reported that: "the year 1816, however, is relatively warm in the Langara reconstruction and was likely associated with an El Niño event".
South Pacific Ocean				
Australia (the main island continent – which was then inhabited by Indigenous peoples and some British settlers)	Probably yes overall: Yes: [Garden] [Allen] [Gergis]	Yes [Garden]	–	Historical records indicated wet weather, gales and floods in Sydney in New South Wales (NSW) in 1816[48,49]. For the larger region of south-eastern Australia, historical records indicate droughts in 1815-16, but the interpretation of this is unclear given that this drought period appears to have started earlier in 1813 and droughts in the 1800s in this region were fairly common. But this study also reported that 1816-17 were particularly wet years in coastal NSW[50]. Gergis et al[51] reported evidence of decreased precipitation in the 1815-1820 period for south-eastern Australia based on proxy palaeoclimate data eg, tree-rings (Fig. 5 in Gergis

Island (as in 1815-17)	Anomal-ous weather/ climate	Adverse impacts on food production	Food insecurity	Comments / sources
	[Brönnimann]<br>[Tierney]<br>No:<br>[Cullen]			et al). But in south-western Australia, reconstructed rainfall from tree-ring data did not show any notable impact (Fig. 5 in: [52]).<br>According to historical reports[48] "cropping and pastoralism briefly suffered" and the impact was more severe in NSW than Tasmania.<br>***Regional analyses:*** In a regional analysis using instrumental data, proxies and modelling, there was decreased precipitation at the NSW site (Fig. 9 in: [9]).<br>A Western Pacific Ocean wide analysis from coral samples (including 3 sample sites in Northern Australia) reported sea surface temperature reductions after the Tambora eruption[31]. This analysis applied to the northern half of Australia's coastline.<br>A regional analysis that modelled temperature in south-eastern Australia (with tree-ring data from Tasmania) estimated temperature declines for the states of Victoria, NSW and South Australia around 1816 (Figs. 3 and 5 in: [53]).<br>***Note:*** Modelling work for other eruptions (ie, Krakatau, 1883; Santa Maria, 1902; Pinatubo, 1991) suggests that the strongest temperature responses are "over more northerly regions than southerly regions of Australia…"[54].
Australia – Tasmania (then named "van Diemen's Land"). It was inhabited by Indigenous peoples and a small level of European settlement.	Yes<br>[Garden]<br>[Allen, 2001, 2017, 2019]	Yes<br>[Garden]	–	Historical records indicate relatively wet and cold weather in Tasmania, with snowfalls being reported at the time[48]. Flooding was also reported in 1816[49] and there was some crop damage reported (albeit fairly minimal and less than in NSW to the north)[48].<br>In a 277 year cool season dam inflow reconstruction using tree-rings in a site in Western Tasmania, the 1816 year had the highest inflows[49]. Statistical analysis also indicated a relationship with such inflows in the year after 6 volcanic eruptions (including Tambora) ($p \leq 0.01$) (see Supporting Information for this study of Tasmania[49]).<br>A study of tree-rings (albeit with no temperature reconstruction) indicated reduced "ring-width index" for samples at four sites around the time of the Tambora eruption (Fig. 2 in: [55]). Another tree-ring study, but with a temperature reconstruction, also shows signs of a Tambora impact (Fig. 5C in: [56]). This more recent tree-ring work probably supersedes the findings of two earlier tree-ring studies that did not provide any clear evidence of a temperature impact[57,58].<br>***Regional analysis:*** A regional analysis that modelled temperature in south-eastern Australia (informed by 3 studies of tree-ring data from Tasmania) estimated temperature declines for Tasmania in 1816 (Figs. 5 and 6 in: [53]).

Island (as in 1815-17)	Anomal-ous weather/ climate	Adverse impacts on food production	Food insecurity	Comments / sources
				Note: Modelling work for other eruptions (as detailed for Australia above), suggests that the strongest temperature responses are "over more northerly regions than southerly regions of Australia, with weakest responses over Tasmania"[54].
Indonesia – Borneo (Kalimantan is the Indonesian portion of Borneo; other parts are East Malaysia and Brunei)	Yes [D'Arrigo] [Brönnimann] [Camelia] [Tierney]	–	–	***Regional analyses:*** A regional analysis[59] using tree-ring data and coral data suggested a drop in sea surface temperatures for this "Indonesian Warm Pool Region" after the eruption (albeit relevant data sites for this time period were all from Java and Lombok). Nevertheless, "an ENSO warm event ~1817 may have contributed to some of the cooling after Tambora".<br>Another regional analysis using instrumental data, proxies and modelling, there was increased precipitation in the north (Fig. 9 [left panel] in: [9]).<br>In another regional analysis, tree-ring data and modelling suggested both a higher and lower PDSI score in different parts of Borneo – but with the lower score (increased drought risk) dominating (Fig. 9 [right panel] in: [9]). Similar results are also presented elsewhere (Fig. 1 in: [42]).<br>Another regional analysis using coral data from Bali along with modelling, concluded that the "Tambora eruption caused a decrease in the surface temperature of -0.4 to -1°C throughout Indonesia in 1816-1817"[60].<br>A Western Pacific Ocean wide analysis from coral samples (with multiple samples within Indonesia) reported sea surface temperature reductions after the Tambora eruption[31].<br>***Note:*** In this analysis we ignored the direct ash fall impacts of the eruption of Tambora in 1815 that impact on the southern part of Kalimantan.
Indonesia – Sulawesi (formerly "Celebes")	Yes [D'Arrigo] [Brönnimann] [Camelia] [Tierney]	–	–	***Regional analyses:*** A regional analysis[59] using tree-ring data and coral data suggested a drop in sea surface temperatures for this "Indonesian Warm Pool Region" after the eruption (albeit relevant data sites for this time period were all from Java and Lombok). Nevertheless, "an ENSO warm event ~1817 may have contributed to some of the cooling after Tambora".<br>In another regional analysis using tree-ring data (including from Sulawesi[41]) and modelling, there were both higher and lower PDSI scores in different parts of Sulawesi (reduced and increased drought risk respectively, albeit more of the latter) (Fig. 9 in: [9]). But another such analysis showed higher PDSI scores (Fig. 1 in: [42]).<br>Another regional analysis using coral data from Bali along with modelling, concluded that the "Tambora eruption caused a decrease in the surface temperature of -0.4 to -1°C throughout Indonesia in 1816-1817[60].

Island (as in 1815-17)	Anomal-ous weather/ climate	Adverse impacts on food production	Food insecurity	Comments / sources
				A Western Pacific Ocean wide analysis from coral samples (with a sample site from Sulawesi) reported sea surface temperature reductions after the Tambora eruption[31]. ***Note:*** In this analysis we ignored the direct ash fall impacts on Sulawesi in 1815. There was no European settlement on Sulawesi in 1816.
New Britain (part of modern day Papua New Guinea [PNG]). There was no European settlement on this island in 1816.	Yes [D'Arrigo] [Tierney]	–	–	***Regional analyses:*** A regional analysis[59] using tree-ring data and coral data suggested a drop in sea surface temperatures for this "Indonesian Warm Pool Region" after the eruption (albeit relevant data sites for this time period were all from Java and Lombok). Nevertheless, "an ENSO warm event ~1817 may have contributed to some of the cooling after Tambora". A Western Pacific Ocean wide analysis from coral samples (including a sample site from Rabaul in New Britain and one in nearby New Ireland) reported sea surface temperature reductions after the Tambora eruption[31].
New Guinea (now both part of modern day PNG and part of Indonesia)	Yes [D'Arrigo] [Tierney]	–	–	As per New Britain (above), but with the Western Pacific Ocean wide analysis including a sample site from Madang in PNG.
New Zealand – North Island (then part of the British Empire)	Probably yes overall: Yes: [Higgins] [Palmer & Ogden] [Norton] [Duncan] [Fowler] No: [Palmer & Xiong] [Xiong]	–	–	Using data from 8 tree species (with samples from both islands), a study by Higgins et al[61] reported that such tree-ring data was sensitive to major volcanic events, with an apparent cooling after Tambora (Fig. S11 in Higgins et al). Earlier studies of tree-ring data also support a climate impact at this time (with data from both North and South Islands[57,62,63] and for North Island samples[64,65]). Other work has showed no clear impact (using mainly North Island samples)[66,67]. In 1816 there was no significant written record of local weather and societal conditions from Indigenous New Zealanders (Māori) or the very small numbers of Europeans present in the country (the first missionary was Samuel Marsden who arrived in 1814).
New Zealand – South Island	Probably yes overall: Yes:	–	–	Various studies of tree-ring data support a climate impact at this time[57,61-63,68]. These studies had both North and South Island data, except for the one by Fenwick[68] which had only South Island data. Another study with tree-ring data from two South Island sites also supported an impact[65] (with an early analysis of one of these same sites also showing an impact[69]). But another South Island tree-ring study had an analysis with a suggestive

Island (as in 1815-17)	Anomal-ous weather/ climate	Adverse impacts on food production	Food insecurity	Comments / sources
	[Higgins] [Palmer] [Norton] [Fenwick] [Duncan] [Cook, 2002, 2006] Both cooling and no effect: [Cook, 2002]			cooling and an analysis indicating no effect (Figs. 3 and 6 respectively in Cook et al)[70]. See also the details for the North Island of New Zealand (above).
Indian Ocean				
Indonesia – Sumatra (part of the British Empire until Dutch rule began again in 1816)	Yes [Brönnimann] [Camelia] [Tierney]	–	–	***Regional analyses:*** A regional analysis using instrumental data, proxies and modelling, there was reduced precipitation in the north (Fig. 9 [left panel] in: [9]).<br>In a regional analysis using tree-ring data (including from neighbouring Java[41]) and modelling, there was a lower PDSI score (higher drought risk) (Fig. 9 in: [9]). The same results are also presented elsewhere (Fig. 1 in: [42]).<br>Another regional analysis using coral data from Bali along with modelling, concluded that the "Tambora eruption caused a decrease in the surface temperature of -0.4 to -1°C throughout Indonesia in 1816-1817[60].<br>An Indian Ocean wide analysis from coral samples (with a sample site on the west coast of Sumatra) reported sea surface temperature reductions after the Tambora eruption[31].<br>***Note:*** In this analysis we ignored the direct ash fall impacts on southern Sumatra in 1815[71]).
Indonesia – Java	Yes [Wilson] [D'Arrigo] [Camelia] [Tierney] [Brönnimann]	No [van Zanden, 2003] [van Zanden, 2010]	No [van Zanden. 2010] [Peper]	Data for Java on annual GDP per capita and consumption per capita from 1815-1817 was relatively stable and so is consistent with no substantive reduction in food production[72]. Also the share of the GDP for agricultural exports and for textile production was relatively stable for the 1815-1820 period (Fig. 10 in: [73]). If there were substantial food shortages it would seem possible that exports would have declined. While rice consumption per capita and the rice-growing area did decline (Fig. 2 in van Zanden 2012) – this was part of a pattern for all of 1815 to 1830, and probably reflects other trends eg, use of agriculture land for other food crops (eg, maize, pulses and tubers) and for export crops (eg, textiles,

Island (as in 1815-17)	Anomal-ous weather/ climate	Adverse impacts on food production	Food insecurity	Comments / sources
				coffee, tea, tobacco, sugar and indigo). While there were poor harvests and famine in Java in the 1840s, other famines in the 1800s were not described"[72]. A demographic study of the 1800s in Java also only reported famines in the 1840s[74].<br>***Regional analyses:*** A regional analysis[59] using tree-ring data (4 sites in Java) and coral data (from nearby Lombok) suggested a drop in sea surface temperatures for this "Indonesian Warm Pool Region" after the eruption. Nevertheless, the authors reiterate that "an ENSO warm event ~1817 may have contributed to some of the cooling after Tambora".<br>Another regional analysis using coral data from Bali along with modelling, concluded that the "Tambora eruption caused a decrease in the surface temperature of -0.4 to -1°C throughout Indonesia in 1816-1817[60].<br>A Western Pacific Ocean wide analysis from coral samples (with multiple samples within Indonesia – including nearby Bali) reported sea surface temperature reductions after the Tambora eruption[31].<br>In a regional analysis using tree-ring data (including from Java[41]) and modelling, there was a lower PDSI score (higher drought risk) (Fig. 9 in: [9]). The same results are also presented elsewhere (Fig. 1 in: [42]).<br>***Note:*** In this analysis we ignored the direct ash fall impacts on Java of the eruption of Tambora in 1815.
Madagascar (the "Kingdom of Madagascar" controlled part of the island; and there was some British imperial influence in one area)	Probably yes overall:<br>Yes [Tierney]<br>No: [Zinke]	–	Probably yes (a reported famine, albeit lacking detail) [Campbell 2005]	A study of coral did not show abnormal coral growth at this time, in marked contrast to a cooling in the late 1600s[75].<br>A famine occurred in 1816 in the province of Betanimena[76] and a famine period spanning the years 1816 to 1822 is shown in a graph of famines in Madagascar (Campbell pp148,[77]). But the specific cause/s of these specific famines is not described by this author (or identified in additional literature searches by us). Of note is that such famines may not necessarily have been related to climatic conditions since some famines in the 1816 to 1896 period in Madagascar were linked to disease outbreaks (eg, smallpox) and to conflict (in addition to droughts and cyclones)[76,77]. Indeed, cyclones were associated with destruction of rice and banana crops in the northeast coast in 1817 and 1894 (Campbell pp150-151,[77]), where the Betanimena province was located. An example of internal conflict was a raid in 1816 in southwest Madagascar that took 2000 slaves and 4000 head of cattle (Campbell p67,[77]). Internal conflict in the 1816 to 1853 period also contributed to famines via scorched earth tactics, the spread of disease by troops, and unprovisioned troops stealing food (Campbell p154,[77]).

Island (as in 1815-17)	Anomal-ous weather/ climate	Adverse impacts on food production	Food insecurity	Comments / sources
				Regional analysis: An Indian Ocean wide analysis from coral samples (with a sample site on the coast of Madagascar and in the Mozambique Channel) reported sea surface temperature reductions after the Tambora eruption[31].
Sri Lanka (then part of the British Empire and called Ceylon)	Yes [Tierney]	–	–	***Regional analysis:*** An Indian Ocean wide analysis from coral samples (albeit with no sample sites around Sri Lanka) reported sea surface temperature reductions after the Tambora eruption[31].
Timor (part of modern East Timor and part of modern Indonesia)	Yes [D'Arrigo] [Camelia] [Tierney]	–	–	***Regional analyses:*** A regional analysis[59] using tree-ring data and coral data suggest a drop in sea surface temperatures for this "Indonesian Warm Pool Region" after the eruption (albeit relevant data sites for this time period were all from Java and Lombok). Nevertheless, "an ENSO warm event ~1817 may have contributed to some of the cooling after Tambora".<br>Another regional analysis using coral data from Bali along with modelling, concluded that the "Tambora eruption caused a decrease in the surface temperature of -0.4 to -1°C throughout Indonesia in 1816-1817[60].<br>A Western Pacific Ocean wide analysis from coral samples (including sample sites around Indonesia and Northern Australia) reported sea surface temperature reductions after the Tambora eruption[31].<br>***Note:*** Some ash reached Timor from the eruption[71] but we ignored the direct ash fall impacts in 1815. In 1816 Timor was being fought over by the Dutch and Portuguese.

References

1 Valler, V., Franke, J., Brugnara, Y. & Brönnimann, S. An updated global atmospheric paleo-reanalysis covering the last 400 years. *Geoscience Data Journal* **9**, 89-107 (2022).

2 Zhang, P.-Y., Wang, W.-C. & Hameed, S. Evidence for anomalous cold weather in China 1815-1817. In: Harington CR, The Year Without a Summer?—World Climate in 1816. Ottawa: Canadian Museum of Nature, 1992 (pp436-447).

3 Chenoweth, M. The early 19th century climate of the Bahamas and a comparison with 20th century averages. *Climatic Change* **40**, 577-603 (1998).

4 Wilson, R. M. Variation of surface air temperatures in relation to El Niño and cataclysmic volcanic eruptions, 1796–1882. *Journal of Atmospheric and Solar-Terrestrial Physics* **61**, 1307-1319 (1999).

5 Dawson, A. G., Hickey, K., McKenna, J. & Foster, D. A 200-year record of gale frequency, Edinburgh, Scotland: possible link with high-magnitude volcanic eruptions. *The Holocene* **7**, 337-341 (1997).

6 Veale, L. & Endfield, G. H. Situating 1816, the 'year without summer', in the UK. *The Geographical Journal* **182**, 318-330 (2016).

7 Post, J. *The Last Great Subsistence Crisis in the Western World*. Baltimore and London: Johns Hopkins University Press, 1977.

8 Behringer, W. Tambora and the Year without a Summer. Cambridge: Polity Press, 2019.

9 Brönnimann, S. & Krämer, D. Tambora and the "Year Without a Summer" of 1816. A Perspective on Earth and Human Systems Science. Geographica Bernensia G90, pp48 (2016).

10 Harington, C. The Year Without a Summer?—World Climate in 1816. Ottawa: Canadian Museum of Nature, 1992 (pp576).

11 Lee, D. & MacKenzie, A. Trans-hemispheric effects of large volcanic eruptions as recorded by an early 19th century diary. *International Journal of Climatology* **30**, 2217-2228 (2010).

12 Briffa, K. & Jones, P. The climate of Europe during the 1810s with special reference to 1816. In: C. R. Harrington, (ed), The Year Without a Summer? World Climate in 1816, Canadian Museum of Nature, 1992, pp372-391.

13 Briffa, K. R., Jones, P. D., Schweingruber, F. H. & Osborn, T. J. Influence of volcanic eruptions on Northern Hemisphere summer temperature over the past 600 years. *Nature* **393**, 450-455 (1998).

14 Schurer, A. *et al.* Disentangling the causes of the 1816 European year without a summer. *Environmental Research Letters* **14**, 094019 (2019).

15 Brugnara, Y. *et al.* A collection of sub-daily pressure and temperature observations for the early instrumental period with a focus on the" year without a summer" 1816. *Climate of the Past* **11**, 1027-1047 (2015).

16 Casty, C., Raible, C. C., Stocker, T. F., Wanner, H. & Luterbacher, J. A European pattern climatology 1766–2000. *Climate Dynamics* **29**, 791-805 (2007).

17 Raible, C. C. *et al.* Tambora 1815 as a test case for high impact volcanic eruptions: Earth system effects. *Climate Change* **7**, 569-589 (2016).

18 Reichen, L. *et al.* A decade of cold Eurasian winters reconstructed for the early 19th century. *Nature Communications* **13**, 2116 (2022).

19 Ogilvie, A. 1816 - a year without a summer in Iceland? In: Harington CR, The Year Without a Summer?—World Climate in 1816. Ottawa: Canadian Museum of Nature, 1992 (pp331-354).

20 Schöne, B. R. *et al.* Climate records from a bivalved Methuselah (Arctica islandica, Mollusca; Iceland). *Palaeogeography, Palaeoclimatology, Palaeoecology* **228**, 130-148 (2005).
21 Murphy, C. *et al.* A 305-year continuous monthly rainfall series for the island of Ireland (1711–2016). *Climate of the Past* **14**, 413-440 (2018).
22 Wilson, C. Workshop on world climate in 1816: a summary and discussion of results. In: Harington CR, The Year Without a Summer?—World Climate in 1816. Ottawa: Canadian Museum of Nature, 1992 (pp523-556).
23 Devor, T. The Explanatory Power of Climate History for the 19th-Century Maritimes and Newfoundland: A Prospectus. *Acadiensis* **43**(2), 57-78 (2014).
24 Newell, J. The climate of the Labrador Sea in the spring and summer of 1816, and comparison with modern analogues. In: C. R. Harrington, (ed), The Year Without a Summer? World Climate in 1816, Canadian Museum of Nature, 1992, pp245-254.
25 American Advocate and Kennebec Advertiser. Distress at Newfoundland Newbury Port. *American Advocate and Kennebec Advertiser* 27 May - 7 June 1817; vol 8, issue 21 pg 3.
26 D'Arrigo, R., Buckley, B., Kaplan, S. & Woollett, J. Interannual to multidecadal modes of Labrador climate variability inferred from tree rings. *Climate Dynamics* **20**, 219-228 (2003).
27 Suffling, R. & Fritz, R. The ecology of a famine: northwestern Ontario in 1815–17. In: C. R. Harrington, (ed), The Year Without a Summer? World Climate in 1816, Canadian Museum of Nature, 1992, pp203-217.
28 Szymczak, S., Joachimski, M., Brauning, A., Hetzer, T. & Kuhlemann, J. A 560 yr summer temperature reconstruction for the Western Mediterranean basin based on stable carbon isotopes from Pinus nigra ssp. laricio (Corsica/France). *Climate of the Past* **8**, 1737–1749 (2012).
29 Villalba, R. & Boninsegna, J. Changes in Southern South American Tree-Ring Chronologies following Major Volcanic Eruptions between 1750 adn 1970. In: Harington CR, The Year Without a Summer?—World Climate in 1816. Ottawa: Canadian Museum of Nature, 1992 (pp493-509).
30 Gao, C., Gao, Y., Zhang, Q. & Shi, C. Climatic aftermath of the 1815 Tambora eruption in China. *Journal of Meteorological Research* **31**(1), 28–38 (2017).
31 Tierney, J. E. *et al.* Tropical sea surface temperatures for the past four centuries reconstructed from coral archives. *Paleoceanography* **30**, 226-252 (2015).
32 Ohyama, M. *et al.* Reconstruction of northeast Asia spring temperature 1784–1990. *Climate of the Past* **9**, 261-266 (2013).
33 Tsukamura, Y. The reconstructed position of the polar frontal zone around Japan in the summer of 1816. In: Harington CR, The Year Without a Summer?—World Climate in 1816. Ottawa: Canadian Museum of Nature, 1992 (pp453-461).
34 Mikami, T. & Tsukamura, Y. The climate of Japan in 1816 as compared to an extremely cool summer climate in 1783. In: Harington CR, The Year Without a Summer?—World Climate in 1816. Ottawa: Canadian Museum of Nature, 1992 (pp462-476).
35 Mikami, T. Climatic variations in Japan reconstructed from historical documents. *Weather* **63**, 190-193 (2008).
36 Aono, Y. & Kazui, K. Phenological data series of cherry tree flowering in Kyoto, Japan, and its application to reconstruction of springtime temperatures since the 9th century. *International Journal of Climatology* **28**, 905-914 (2008).

37 Aono, Y. Cherry blossom phenological data since the seventeenth century for Edo (Tokyo), Japan, and their application to estimation of March temperatures. *International Journal of Biometeorology* **59**, 427-434 (2015).
38 Mikami, T. Long term variations of summer temperatures in Tokyo since 1721. *Geographical Reports of Tokyo Metropolitan University* **31**, 157-165 (1996).
39 Bassino, J.-P.
40 Saito, O. The frequency of famines as demographic correctives in the Japanese past. In: Famine Demography: Perspectives from the Past and Present (T. Dyson and C. O'Grada, eds.) Oxford: Oxford University Press, 2002. pp. 218-239.
41 Cook, E. R. *et al.* Asian monsoon failure and megadrought during the last millennium. *Science* **328**, 486-489 (2010).
42 Fasullo, J. *et al.* The amplifying influence of increased ocean stratification on a future year without a summer. *Nature Communications* **8**, 1-10 (2017).
43 Inoue, M. *et al.* A 228-year coral record in the Philippines reveals volcanic cooling in the nineteenth century and globally coherent warming in the late twentieth century. (2022).
44 Warren, J. F. Typhoons and droughts: Food shortages and famine in the Philippines since the seventeenth century. *International Review of Environmental History* **4**, 27-44 (2018).
45 Ramos, R., Goodkin, N. & Fan, T. Y. Coral records at the northern edge of the Western Pacific warm pool reveal multiple drivers of sea surface temperature, salinity, and rainfall variability since the end of the Little Ice Age. *Paleoceanography and Paleoclimatology* **35**, e2019PA003826 (2020).
46 Parker, M. Investigating the possibility of a relationship between volcanic eruptions and tree growth in Canada (1800-1899). *Syllogeus* **55**, 249-264 (1985).
47 Edge, D. C. *et al.* A Multicentennial Proxy Record of Northeast Pacific Sea Surface Temperatures From the Annual Growth Increments of Panopea generosa. *Paleoceanography and Paleoclimatology* **36**, e2021PA004291 (2021).
48 Garden, D. A Wet and Cold El Niño: the Tambora volcano's impact in the Australian colonies. *Journal of the Royal Australian Historical Society* 2021;107(2):179-201.
49 Allen, K. *et al.* A 277 year cool season dam inflow reconstruction for Tasmania, southeastern Australia. *Water Resources Research* **53**, 400-414 (2017).
50 Fenby, C. & Gergis, J. Rainfall variations in south-eastern Australia part 1: Consolidating evidence from pre-instrumental documentary sources, 1788–1860. *International Journal of Climatology* **33**, 2956-2972 (2013).
51 Gergis, J. *et al.* On the long-term context of the 1997–2009 'Big Dry' in South-Eastern Australia: insights from a 206-year multi-proxy rainfall reconstruction. *Climatic Change* **111**, 923-944 (2012).
52 Cullen, L. E. & Grierson, P. F. Multi-decadal scale variability in autumn-winter rainfall in south-western Australia since 1655 AD as reconstructed from tree rings of Callitris columellaris. *Climate Dynamics* **33**, 433-444 (2009).
53 Allen, K. *et al.* Tree-ring reconstructions of cool season temperature for far southeastern Australia, 1731–2007. *Climate Dynamics* **53**, 569-583 (2019).
54 Harvey, P. J. & Grab, S. W. Australian sub-regional temperature responses to volcanic forcing: A critical analysis using CMIP5 models. *International Journal of Climatology* (2022).
55 Allen, K., Cook, E., Francey, R. & Michael, K. The climatic response of Phyllocladus aspleniifolius (Labill.) Hook. f in Tasmania. *Journal of Biogeography* **28**, 305-316 (2001).

56 Cook, E., Buckley, B., D'arrigo, R. & Peterson, M. Warm-season temperatures since 1600 BC reconstructed from Tasmanian tree rings and their relationship to large-scale sea surface temperature anomalies. *Climate Dynamics* **16**, 79-91 (2000).
57 Palmer, J. & Ogden, J. Tree Ring Chronologies from Endemic Australian and New Zealand Conifers 1800-30. In: Harington CR, The Year Without a Summer?—World Climate in 1816. Ottawa: Canadian Museum of Nature, 1992 (pp510-515).
58 Cook, E. *et al.* Climatic change over the last millennium in Tasmania reconstructed from tree-rings. *The Holocene* **2**, 205-217 (1992).
59 D'Arrigo, R. *et al.* The reconstructed Indonesian warm pool sea surface temperatures from tree rings and corals: Linkages to Asian monsoon drought and El Nino–Southern Oscillation. *Paleoceanography* **21** (2006).
60 Camelia, H., Herho, S., Syahputra, M. & Suwarman, R. Assessing the impact of 1815 Tambora volcanic eruption on surface temperature and precipitation variabilities in Indonesia using MPI-ESM simulation outputs.
61 Higgins, P. A., Palmer, J. G., Turney, C. S., Andersen, M. S. & Johnson, F. Do Southern Hemisphere tree rings record past volcanic events? A case study from New Zealand. *Climate of the Past* **18**, 1169-1188 (2022).
62 Norton, D. New Zealand temperatures, 1800-30. In: Harington CR, The Year Without a Summer?—World Climate in 1816. Ottawa: Canadian Museum of Nature, 1992 (pp516-520).
63 Duncan, R. P., Fenwick, P., Palmer, J. G., McGlone, M. S. & Turney, C. S. Non-uniform interhemispheric temperature trends over the past 550 years. *Climate Dynamics* **35**, 1429-1438 (2010).
64 Fowler, A. M. *et al.* Multi-centennial tree-ring record of ENSO-related activity in New Zealand. *Nature Climate Change* **2**, 172-176 (2012).
65 Cook, E. R. *et al.* Millennia-long tree-ring records from Tasmania and New Zealand: A basis for modelling climate variability and forcing, past, present and future. *Journal of Quaternary Science* **21**, 689-699 (2006).
66 Xiong, L. & Palmer, J. G. Reconstruction of New Zealand temperatures back to AD 1720 using Libocedrus bidwillii tree-rings. *Climatic Change* **45**, 339-359 (2000).
67 Palmer, J. G. & Xiong, L. New Zealand climate over the last 500 years reconstructed from Libocedrus bidwillii Hook. f. tree-ring chronologies. *The Holocene* **14**, 282-289 (2004).
68 Fenwick, P. Reconstruction of past climates using pink pine (Halocarpus biformus) tree-ring chronologies [PhD thesis]. Christchurch, New Zealand: Soil Plant and Ecological Sciences, Linclon University, 2003. https://researcharchive.lincoln.ac.nz/handle/10182/2141.
69 D'Arrigo, R. *et al.* Tree-ring records from New Zealand: long-term context for recent warming trend. *Climate Dynamics* **14**, 191-199 (1998).
70 Cook, E. R., Palmer, J. G., Cook, B. I., Hogg, A. & D D'Arrigo, R. A multi-millennial palaeoclimatic resource from Lagarostrobos colensoi tree-rings at Oroko Swamp, New Zealand. *Global and Planetary Change* **33**, 209-220 (2002).
71 de Jong Boers, B. Mount Tambora in 1815: A Volcanic Eruption in Indonesia and Its Aftermath. *Indonesia* **60**, 37–60 (1995).
72 van Zanden, J. Colonial state formation and patterns of economic development in Java, 1800–1913. *Economic History of Developing Regions* **25**, 155-176 (2010).
73 van Zanden, J. L. Economic growth in Java 1815-1939 The reconstruction of the historical national accounts of a colonial economy. Unpublished Maddison-Project Working Paper WP-3 (2012).

74 Peper, B. Population growth in Java in the 19th century: A new interpretation. *Population Studies* **24**, 71-84 (1970).
75 Zinke, J., Dullo, W.-C., Heiss, G. & Eisenhauer, A. ENSO and Indian Ocean subtropical dipole variability is recorded in a coral record off southwest Madagascar for the period 1659 to 1995. *Earth and Planetary Science Letters* **228**, 177-194 (2004).
76 Campbell, G. The State and Pre-Colonial Demographic History: The Case of Nineteenth-Century Madagascar1. *The Journal of African History* **32**, 415-445 (1991).
77 Campbell, G. *An economic history of imperial Madagascar, 1750-1895: the rise and fall of an island empire*. Vol. 106 (Cambridge University Press, 2005).
